# Associations between Adherence to Four A Priori Dietary Indexes and Cardiometabolic Risk Factors among Hyperlipidemic Patients

**DOI:** 10.3390/nu13072179

**Published:** 2021-06-24

**Authors:** Xiaoli Gao, Zezhong Tian, Dan Zhao, Kongyao Li, Yimin Zhao, Lin Xu, Xu Wang, Die Fan, Xilin Ma, Wenhua Ling, Huicui Meng, Yan Yang

**Affiliations:** 1 School of Public Health (Shenzhen), Sun Yat-sen University, Shenzhen 518106, China; gaoxli3@mail3.sysu.edu.cn (X.G.); tianzzh@mail2.sysu.edu.cn (Z.T.); zhaod39@mail2.sysu.edu.cn (D.Z.); liky33@mail2.sysu.edu.cn (K.L.); zhaoym26@mail.sysu.edu.cn (Y.Z.); xulin35@mail2.sysu.edu.cn (L.X.); maxlin3@mail3.sysu.edu.cn (X.M.); menghc@mail.sysu.edu.cn (H.M.); 2 Guangdong Provincial Key Laboratory of Food, Nutrition and Health, Guangzhou 510080, China; lingwh@mail.sysu.edu.cn; 3 Guangdong Engineering Technology Center of Nutrition Transformation, Guangzhou 510080, China; 4 Department of Nutrition, School of Public Health, Sun Yat-sen University, Guangzhou 510080, China; wangxu25@mail2.sysu.edu.cn (X.W.); fand3@mail2.sysu.edu.cn (D.F.)

**Keywords:** diet balance index, Chinese healthy eating index, Mediterranean diet score, dietary approaches to stop hypertension score, cardiometabolic disorders, dyslipidemia

## Abstract

Little is known about which currently available a priori dietary indexes provide best guidance for reducing cardiometabolic risk factors (CMRF) among hyperlipidemic patients. This study was designed to compare the associations between four a priori dietary indexes, including Diet Balance Index (DBI-16), Chinese Healthy Eating Index (CHEI), Mediterranean Diet Score (MDS) and Dietary Approaches to Stop Hypertension (DASH) and CMRF among hyperlipidemic patients. A total of 269 participants were enrolled into the cross-sectional study. DBI-16, CHEI, MDS, and DASH scores were calculated using established methods. CMRF was measured using standard methods. DBI-total scores (DBI-TS) were inversely associated with triglyceride concentrations and TC:HDL-C ratio, and positively associated with HDL-C and ApoA1 concentrations (all *p* < 0.05), while the results for DBI-low bound scores (DBI-LBS) were opposite. DBI-high bound scores (DBI-HBS) and DASH scores were positively and inversely associated with glucose concentrations, respectively (both *p* < 0.05). Higher diet quality distance (DQD) was positively associated with higher TC, LDL-C and ApoB concentrations, and TC:HDL-C and LDL-C:HDL-C ratios, and lower HDL-C and ApoA1 concentrations and ApoA1:ApoB ratio (all *p* < 0.05). CHEI scores were inversely associated with triglyceride concentrations (*p* = 0.036). None of the dietary indexes was associated with blood pressures. DBI-16 provided most comprehensive evaluations of the overall diet quality and balance for optimizing cardiometabolic health among hyperlipidemic individuals.

## 1. Introduction

Cardiometabolic disorders, including cardiovascular disease and type 2 diabetes (T2D), are major noncommunicable diseases (NCDs) in China and globally [1,2]. Observational and interventional studies have consistently reported that dysregulation in lipid and lipoprotein profiles, glucose homeostasis biomarkers, and blood pressures contribute to increased morbidity and mortality of cardiometabolic disorders [3,4,5]. Hyperlipidemia is an independent risk factor for cardiometabolic disorders [6,7]. Approximately 40% Chinese adults are suffering from hyperlipidemia, and early prevention against cardiometabolic risk factors in hyperlipidemic patients is required [8,9,10]. Dietary interventions are fundamental and modifiable approaches for cardiometabolic disorder prevention via the regulations of cardiometabolic risk factors [11]. Despite the research focus on associations between individual nutrients or food components and cardiometabolic risk factors in prior studies [12,13], an increasing number of studies have recognized the synergistic and inter-related effects of different dietary and food components on cardiometabolic health because dietary and food components are never consumed in isolation [14,15].

Dietary patterns, which describe the overall quality of diet, have been applied as emerging approaches to investigate the interplay between diet and cardiometabolic health [16]. To assess individuals’ adherence to specific dietary patterns, several a priori dietary indexes have been created [15], such as the Healthy Eating Index (HEI), Diet Quality Index (DQI), Mediterranean Diet Score (MDS), and Dietary Approaches to Stop Hypertension (DASH) score [17,18,19]. These a priori dietary indexes are simple to calculate, and the results are objective and easy to interpret. However, questions have been raised as to whether they could be used to guide food choices aimed at reducing cardiometabolic risk among different populations. Although inverse associations between HEI, DQI, MDS, and DASH scores and cardiometabolic risk have been documented in both observational and interventional studies [20,21,22,23], data among Chinese populations are limited, especially in hyperlipidemic patients.

With reference to the methods of DQI, a new dietary quality assessment index, Diet Balance Index (DBI-16), was created based on the recommended intake of the balanced diet and to reveal both inadequate and excessive food intake among Chinese populations [24]. Previous studies have found that unfavorable dietary quality evaluated on the basis of DBI criteria are associated with unfavorable blood glucose and HDL-C concentrations [25] and higher prevalence of prediabetes [26] among Chinese adults. However, the associations between DBI-16 scores and cardiometabolic risk factors among hyperlipidemic patients are unclear. On the basis of HEI, Chinese Healthy Eating Index (CHEI) was developed according to the most recent Dietary Guidelines for Chinese (DGC-2016) and the Chinese Food Pagoda (CFP) [27] to assess the overall diet quality in Chinese populations. To date, little is known about whether a priori dietary indexes, especially DBI-16, CHEI, MDS and DASH score, are associated with cardiometabolic risk factors among Chinese hyperlipidemic patients, and little attention has been given to the comparisons among these indexes. This missing information limits efforts to refine dietary guidance intended to reduce cardiometabolic risk in Chinese populations at elevated risk for cardiometabolic disorders.

The aim of this study was to assess and compare the associations between four a priori dietary indexes, including DBI-16, CHEI, MDS, and DASH score and cardiometabolic risk factors (lipid and lipoprotein profile, glucose homeostasis biomarkers and blood pressures) and identify the relatively optimal a priori dietary index for evaluating diet quality and balance in Chinese hyperlipidemic patients in a cross-sectional setting.

## 2. Materials and Methods

### 2.1. Study Population

This is an ancillary study and post-hoc analysis of our previous study, which is a randomized double-blinded placebo-controlled trial investigating the effect of anthocyanin supplementation on lipid profiles, oxidative stress, and inflammation in hyperlipidemic patients [28,29]. To be consistent with the parent study, the current study selected hyperlipidemic patients. Study participants (*n* = 269 Chinese men and women, 35–65 years old, resident in Guangzhou for at least the past 10 years) were recruited from Guangzhou, China, and had hyperlipidemia (defined as comprising at least two of the following four criteria: fasting plasma concentrations of triglyceride ≥ 1.70 mmol/L, total cholesterol [TC] ≥ 5.20 mmol/L, low-density lipoprotein cholesterol [LDL-C] ≥ 3.12 mmol/L, high-density lipoprotein cholesterol [HDL-C] ≤ 0.91 mmol/L) according to the “*2016 Chinese Guideline for the Management of Dyslipidemia in Adults*”. Exclusion criteria included known chronic diseases (including type 2 diabetes, myocardial infarction, stroke, untreated hypertension, cancer, and liver and kidney dysfunction); acute and chronic infectious diseases, trauma or surgery; use of hormonal therapies; use of medications known to influence lipid metabolism (including statins, fibrates, and bile acid sequestrants) within the past six months; use of anti-inflammatory or antibiotic drugs within the past three months; use of vasomotor function drugs within the past three months; taking phytochemicals (such as anthocyanins and grape seed extract) or other dietary supplements within the past two months and pregnant or lactating women [29]. The study was conducted in accordance with the Declaration of Helsinki guidelines. All methodologies, protocols, and procedures were approved by the ethics committee of School of Public Health, Sun Yat-sen University ((2019) No. 134), and written informed consents were obtained from all study participants.

### 2.2. Sample Size Estimation

Sample size estimation was conducted using PASS 15.0 software (NCSS, Kaysville, UT, USA). The sample size was determined based on the results of a Chinese cardiovascular research study [8], which has reported that the prevalence of dyslipidemia in Chinese adults is approximately 40%. With the use of an allowable error of 0.06 (0.2-fold prevalence) and type Ⅰ error of 0.05 (2 tail), the estimated sample size was 269 participants.

### 2.3. Recruitment and Screening

Volunteers who were interested in the study advertisements were contacted via telephone to assess potential eligibility, and if they met inclusion criteria, were invited for an onsite visit at the School of Public Health of Sun Yat-sen University (SYSU) to learn specific information about the study and attend a pre-screening test. During the in-person visit, fasting venous blood samples were collected and lipid and lipoprotein profiles were measured to confirm eligibility. Volunteers were included in the study and signed informed consent forms if they qualified according to the pre-determined criteria. A total of 601 volunteers received pre-screening tests and 269 participants were enrolled into the study (see Figure S1—Online Supplementary Figure for participant flow).

### 2.4. Assessment of Dietary Intake Information

Dietary intake information was assessed using a validated interviewer-administered 79-item semi-quantitative food frequency questionnaire (FFQ) [30,31]. Participants answered two questions about each food item: their usual frequency (daily, weekly, monthly, yearly, never) of consuming specific foods or beverages over the past 1 year; and the amount of consumption at each time. The average daily intake of each food item was calculated by multiplying the intake frequency of each food per day by the amount consumed at each time. Nutrients from each food item were calculated based on the 2018 Chinese Food Composition Table (Standard Edition) [32].

### 2.5. Calculation of a Priori Dietary Indexes

DBI-16 [26], CHEI [27], MDS [17] and DASH score [19,33] were calculated with the dietary intake information collected from FFQ. The DBI-16 has eight food components including cereals, vegetables and fruits, beans and dairy products, animal foods, empty energy foods, condiments, diet variety, and water. A score of 0 is given when the food intake meets the recommendation of the dietary guidelines, and the negative or positive scores indicate that the actual intake levels are insufficient or excessive compared to the recommended levels, respectively (Supplementary Table S1). DBI-16 has four indicators of dietary quality, including total score (TS), low bound scores (LBS), high bound scores (HBS), and diet quality distance (DQD) [24]. DBI-TS, which ranges from −72 to 44, is calculated by summing all scores of eight food groups to reflect the overall diet quality [34]. If DBI-TS is negative, the dietary intake tends to be insufficient; and if DBI-TS is positive, the dietary intake tends to be excessive. A value of 0 of DBI-TS indicates a balanced diet, without insufficient or excessive intakes. DBI-LBS is the sum of absolute values of negative scores, indicating the degree of insufficient dietary intake. DBI-LBS is the sum of absolute values of positive scores, indicating the degree of excessive dietary intake. The DQD is defined as the sum of DBI-LBS and DBI-HBS and reveal whether the individual’s food intake is balanced. The ranges of DBI-LBS, DBI-HBS, and DQD are 0–72, 0–44, and 0–96, respectively. A score of 0 indicates excellent dietary intake (no problems), a score that is less than 20% of the total score indicates good dietary intake (almost no problems), a score that is 20–40% of the total score indicates acceptable dietary intake (moderate level of problems), a score that is 40–60% of total score indicates poor dietary intake (moderate level of problems), and a score greater than 60% of the total score indicates the worst dietary intake (high level of problems) [26,35].

CHEI has a continuous scoring system, containing 17 food components ranging from 0–100. Twelve of the 17 components evaluate the adequacy of diet (including total grains, whole grains and mixed beans, tubers, total vegetables, dark vegetables, fruits, dairy, soybeans, fish and seafoods, poultry, eggs, seeds and nuts), and the other five components assess the limitation of diet (red meat, cooking oils, sodium, added sugars, alcohols). Higher intakes of adequacy food components resulted in higher scores, whereas higher intakes of limitation food components resulted in lower scores (Supplementary Table S2).

The MDS is a scale based on the intake of 14 questions to indicate the degree of adherence to the traditional Mediterranean diet. Meat and dairy product intake less than the median of all study participants received 1 point, and greater than the median received 0 point [17]. For all other food items, intakes above the median received 1 point and 0 point otherwise. Possible MDS scores ranged from 0 (minimal adherence to traditional Mediterranean diet) to 14 (maximal adherence) [18] (Supplementary Table S3).

The DASH score was composed of eight food groups, including grains, vegetables, fruits, dairy products, meat, nuts/seeds/legumes, fats/oils and sweets, which are emphasized in the DASH diet [36]. For each food group [37], a maximum score of 10 could be achieved when the intakes meet recommendation. If lower intakes are favored by DASH diet, reverse scoring is applied [19,33] (Supplementary Table S4). Adherence to the DASH diet is based on the overall score, ranging from 0 to 80.

### 2.6. Assessment of Cardiometabolic Risk Factors

Systolic and diastolic blood pressures of participants were measured twice using an automated blood pressure monitor after a quiet rest for at least 5 min. The average of two measurements was calculated and recorded. Venous blood samples were collected between 8:00 am and 9:00 am following an overnight fast (8–10 h). Venous blood samples were immediately centrifuged at 3000 rpm for 15 min at 4 ℃ for serum collection. Enzymatic methods were used to determine the concentrations of serum triglyceride, TC, LDL-C and HDL-C on the Cobas 8000 c702 automated assay analyzer (Roche Diagnostics, Basel, Switzerland). The concentrations of Apolipoprotein A1 (ApoA1) and Apolipoprotein B (ApoB) were determined by immunonephelometry using Cobas 8000 c702 automated assay analyzer (Roche Diagnostics, Basel, Switzerland). Fasting blood glucose concentrations were measured with whole blood using the Cobas c311 automated assay analyzer (Roche Diagnostics, Basel, Switzerland). Concentrations of glycated hemoglobin A1c (HbA1c) were determined by cation-exchange high-pressure liquid chromatography (Bio-Rad Laboratories, Hercules, CA, USA).

### 2.7. Covariates

Data on sociodemographic and lifestyle information, including marital status, education, smoking status, and employment, were collected using validated questionnaires. Height was measured to the nearest 0.1 cm using a portable stadiometer, and weight was measured with an electronic weighing scale to the nearest 0.1 kg. Body mass index (BMI) was calculated by dividing the weight (kg) by height squared (m^2^). Waist circumference was measured at the narrowest part of the waist using a tape measure to the nearest 0.1 cm. Measurements of height, weight, and waist circumference were repeated twice, and the average of two independent measurements was calculated and recorded. Physical activity status was measured using the International Physical Activity Questionnaire (IPAQ) [38,39], and outcome was presented as metabolic equivalent task (MET) hours per week (MET-h/week).

### 2.8. Statistical Analyses

Data were analyzed using SPSS statistical software (v19.0, SPSS, Inc., Chicago, IL, USA) and R 4.0.2 software (Bell Laboratories, Murray Hill, NJ, USA, http://www.R-project.org/, accessed on 16 June 2021) with rms and risk Regression packages. Continuous variables were presented as mean ± standard deviation (SD), and categorical variables were presented as *n* (%). Data were tested for normality prior to statistical analyses. Differences in sociodemographic and lifestyle characteristics between female and male participants were compared using the 2-sample t test for continuous variables or chi-square for categorical variables. Differences in food and macronutrient intake and cardiometabolic risk factors between female and male participants were compared using 2-sample t test. Multivariable linear regression models were used to determine the associations between the four a priori dietary indexes (DBI-16, CHEI, MDS, and DASH score) and cardiometabolic risk factors (lipid and lipoprotein profiles, glucose homeostasis biomarkers and blood pressures). In model 1, the data were adjusted for potential confounders, including age (continuous as y), sex (male or female), and BMI (continuous as kg/m^2^). Model 2 was fully adjusted model and included model 1 plus additional sociodemographic and lifestyle confounders, including cigarette smoking (yes or no), education status (not attending school, primary school, junior high school, high school/secondary school, college, or Bachelor’s degree and above), physical activity (continuous as MET-h/week), and total energy intake (continuous as kcal/d). Subgroup analyses were performed with the same multivariable linear regression models in both female and male participants, respectively.

If significant associations were observed between at least two a priori dietary indexes and specific cardiometabolic risk factors, each variable of the cardiometabolic risk factor was dichotomized by corresponding mean value, and logistic regression models with receiver operating characteristic curve (ROC) were used to provide a conclusive mean for the comparison among four a priori dietary indexes. For model prediction performance, discrimination and calibration were evaluated. Discrimination represented the ability of the dietary indexes to differentiate between patients who did and did not have a high level of cardiometabolic risk factors. The measurement of discrimination is quantified by calculating the area under the ROC statistic. Calibration represented the agreement between predicted probabilities from the models and observed outcomes. We used the Hosmer-Lemeshow test to statistically determine the extent of agreement between the predicted probabilities and observed outcomes [40]. Furthermore, the overall accuracy of predictions were measured by Brier score, which denoted the observed outcome and the prediction in a dataset of 269 participants [41]. For model validation, internal validation was adopted using a bootstrapping method with 1,000 bootstrap resamples of 269 participants. Statistical significance was accepted at *p* < 0.05.

## 3. Results

### 3.1. Characteristics of the Study Participants

Study participants were middle-aged adults with mean age of 58 ± 8 years; 75.1% were females (Table 1). Approximately half of the participants (50.9%) were overweight or obese and 44.2% had central obesity. Female participants had lower body weights, heights, BMI and waist circumferences, lower percentage of Bachelor’s degree or postgrad, smokers and full-time employment, and higher levels of physical activity compared to male participants (all *p* < 0.05) (Table 1).

### 3.2. Cardiometabolic Risk Factor Levels of Participants

By design, all participants were hyperlipidemic patients. About half of participants had hypertriglyceridemia (47.2%), approximately 90% of participants had hypercholesterolemia and elevated LDL-C (91.8% and 89.2%, respectively), and only 7.4% of participants had low HDL-C (Table 2). The average concentrations of fasting blood glucose and HbA1c were 5.4 mmol/L and 5.8%, respectively. The average systolic and diastolic blood pressures were 115.1 mm Hg and 73.2 mm Hg, respectively, and 7.4% of participants had hypertension. In comparison to male participants, female participants had significantly higher fasting serum concentrations of TC, LDL-C, HDL-C, ApoA1, and ApoA1:ApoB ratio, and lower fasting serum triglyceride concentrations, TC:HDL-C and LDL-C:HDL-C ratios and blood pressures (all *p* < 0.05) (Table 2).

### 3.3. Daily Dietary and Macronutrient Intake of Participants

The daily dietary and macronutrient intake of participants are presented in Table 3. The intakes of dairy, soybean, and related products were lower than the recommended amounts in the Dietary Guidelines for Chinese (DGC-2016), while the intake of other food groups met recommendation. Compared with male participants, female participants had significantly lower total grains and alcohol consumption and higher eggs and dairy and dairy product consumption (all *p* < 0.05) (Table 3). The intake of other food groups were similar between female and male participants (Table 3).

Participants consumed an average of 1643.4 kcal energy and 12.5 g soluble fibers per day (Table 3). Dietary carbohydrate, fat, and protein provided 44.2%, 41.1%, and 17.3% of total energy, respectively (Table 3). The %E from total and saturated fats exceed recommendation levels based on DGC-2016. Male participants had greater daily total energy intake (*p* < 0.001) and less soluble fiber intake (*p* = 0.031) in comparison to female participants (Table 3). No significant differences in %E of macronutrients were observed between female and male participants (Table 3).

### 3.4. Distribution of a Priori Dietary Index Scores among Participants

Based on DBI-TS, the percentage of participants with excessive, excellent, and insufficient dietary intakes was 59.9%, 2.6% and 37.5%, respectively (Table 4). When the diet quality was evaluated with DBI-LBS, DBI-HBS, and DQD, none of the participants had excellent dietary intakes, and the percentage of good dietary intakes was 50.2%, 9.3%, and 97.4%, respectively (Table 4). Almost half of the participants (47.2%) had poor dietary intakes with DBI-HBS evaluation (Table 4). There were no significant differences in distribution of DBI-16 scores among female and male participants (Table 4).

Based on CHEI score, 88.5% participants had high diet quality (Table 4). However, more than 90% participants had low and medium MDS and DASH scores, indicating low adherence rates to both Mediterranean and DASH diets (Table 4). There were no significant differences in the distribution of CHEI, MDS, and DASH scores between female and male participants (Table 4).

### 3.5. Associations between a Priori Dietary Index Scores and Cardiometabolic Risk Factors

In Model 1, there were significant associations between both DBI-TS and DBI-LBS scores and fasting serum concentrations of triglyceride, HDL-C and ApoA1 and TC:HDL-C ratio (all *p* < 0.05) (Table 5). In fully adjusted models (Model 2), DBI-TS scores were inversely associated with fasting serum triglyceride concentrations (β = −0.024 mmol/L, 95% CI: −0.043 to −0.005, R^2^ = 13.3%; *p* = 0.012) and TC:HDL-C ratio (β = −0.023, 95% CI: −0.039 to −0.007, R^2^ = 16.4%; *p* = 0.004), and positively associated with fasting serum concentrations of HDL-C (β = 0.010 mmol/L, 95% CI: 0.004 to 0.015, R^2^ = 28.8%, *p* < 0.001) and ApoA1 (β = 0.005 g/L, 95% CI: 0.002 to 0.008, R^2^ = 25.6%, *p* = 0.002) (Table 5). In contrast, DBI-LBS scores were positively associated with fasting serum triglyceride concentrations (β = 0.032 mmol/L, 95% CI: 0.008 to 0.055, R^2^ = 13.5%, *p* = 0.009), TC:HDL-C ratio (β = 0.034, 95% CI: 0.014 to 0.054, R^2^ = 17.1%, *p* = 0.001) and LDL-C:HDL-C ratio (β = 0.020, 95% CI: 0.005 to 0.035, R^2^ = 11.2%, *p* = 0.011), and inversely associated with fasting serum concentrations of HDL-C (β = −0.013 mmol/L, 95% CI: −0.020 to −0.006, R^2^ = 29.1%, *p* < 0.001) and ApoA1 (β = −0.006 g/L, 95% CI: −0.011 to −0.002, R^2^ = 25.4%, *p* = 0.002) in fully adjusted models (Model 2) (Table 5).

In Model 1, there were significant associations between DBI-HBS scores and fasting blood glucose concentrations and HbA1c (all *p* < 0.05) (Table 5). However, in fully adjusted models (Model 2), DBI-HBS scores were only positively associated with fasting blood glucose concentrations (β = 0.032 mmol/L, 95% CI: 0.003 to 0.062, R^2^ = 8.2%, *p* = 0.033) (Table 5).

In Model 1, there were significant associations between DQD scores and fasting serum concentrations of TC, LDL-C, HDL-C, ApoB and HbA1c and TC:HDL-C ratio, LDL-C:HDL-C ratio, ApoA1:ApoB ratio (all *p* < 0.05) (Table 5). Following further adjusting for lifestyle and dietary factors (Model 2), higher DQD scores were positively associated with higher fasting serum concentrations of TC (β = 0.040 mmol/L, 95% CI: 0.003 to 0.078, R^2^ = 13.3%, *p* = 0.035), LDL-C (β = 0.052 mmol/L, 95% CI: 0.012 to 0.092, R^2^ = 12.1%, *p* = 0.010) and ApoB (β = 0.017 g/L, 95% CI: 0.007 to 0.027, R^2^ = 10.1%, *p* = 0.001), and TC:HDL-C ratio (β = 0.068, 95% CI: 0.023 to 0.114, R^2^ = 16.5%, *p* = 0.003) and LDL-C:HDL-C ratio (β = 0.071, 95% CI: 0.037 to 0.104, R^2^ = 14.5%, *p* < 0.001), and lower concentrations of HDL-C (β = −0.020 mmol/L, 95% CI: −0.036 to −0.005, R^2^ = 27.1%, *p* = 0.010) and ApoA1 (β = −0.011 g/L, 95% CI: −0.021 to −0.002, R^2^ = 24.3%, *p* = 0.019) and ApoA1:ApoB ratio (β = −0.027, 95% CI: −0.041 to −0.014, R^2^ = 13.6%, *p* < 0.001) (Table 5). Subgroup analyses revealed these associations were mainly attributable to female participants, and there was no significant association between DQD and any cardiometabolic risk factors in male participants (Supplementary Tables S5 and S6).

The CHEI scores were only inversely associated with fasting serum triglyceride concentrations (β = −0.018 mmol/L, 95% CI: −0.035 to −0.001, R^2^ = 12.8%, *p* = 0.036) in fully adjusted models (Model 2) (Table 6). In Model 1, there were significant inverse associations between MDS scores and fasting plasma HbA1c concentrations, and the associations were no longer significant in fully adjusted models (Model 2) (Table 6). In both Model 1 and 2, the DASH scores were inversely associated with fasting blood glucose concentrations (model 2: β = −0.017 mmol/L, 95% CI: −0.033 to 0, R^2^ = 8.0%, *p* = 0.046) (Table 6). In fully adjusted models (Model 2), there was no significant association between CHEI, MDS, and DASH scores and cardiometabolic risk factors in both female and male participants (Supplementary Tables S7 and S8). None of the a priori dietary index scores was associated with blood pressures (Table 5 and Table 6).

### 3.6. Comparison of the Associations between a Priori Dietary Index Scores and Cardiometabolic Risk Factors

**Figure 1:**
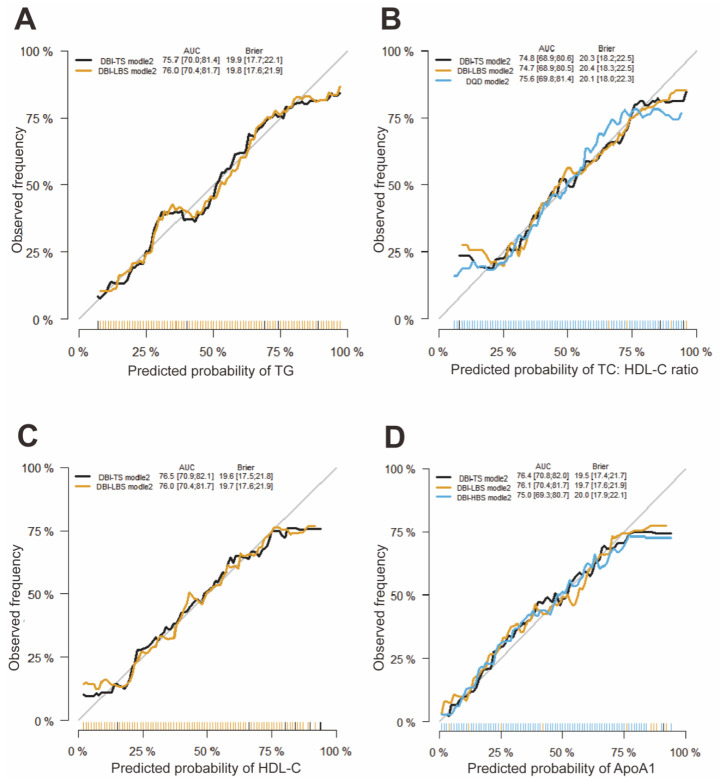
Receiver operating characteristic curve of the logistic regression models. The predicted probability of TG (**A**), TC:HDL-C ratio (**B**), HDL-C (**C**), and ApoA1 (**D**) were analyzed by logistic regression model adjusted for potential confounders, including age, sex, BMI, cigarette smoking, education status, physical activity and total energy intake. The validation was performed with the dataset of 269 participants. AUC and Brier score were expressed as the point estimates and 95% confidence intervals.

**Figure 2:**
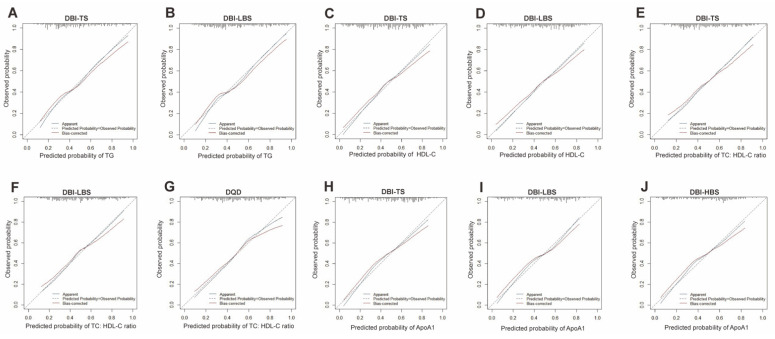
Calibration curve of the logistic regression models. For model validation, internal validation was adopted using a bootstrapping method with 1000 bootstrap resamples of 269 participants for Bias-corrected calibration curves. The apparent calibration curves were performed with the dataset of 269 participants. The predicted probabilities of TG ((**A**): DBI-TS, (**B**): DBI-LBS), HDL-C ((**C**): DBI-TS, (**D**): DBI-LBS), TC: HDL-C ((**E**): DBI-TS, (**F**): DBI-LBS, (**G**): DQD) and ApoA1 ((**H**): DBI-TS, (**I**): DBI-LBS, (**J**): DBI-HBS) by the logistic regression model conforms well to the observed probability. In the plot, all apparent calibration curves and Bias-corrected calibration curves are consistent with and the ideal lines (Predicated Probability = Observed Probability).

In fully adjusted linear regression models (Model 2), DBI-TS and CHEI scores were inversely associated with fasting serum triglyceride concentrations, and DBI-LBS scores were positively associated with fasting serum triglyceride concentrations (all *p* < 0.05) (Table 5). Logistic regression models were conducted to further provide a conclusive mean for the comparison among associations between DBI-TS, DBI-LBS, and CHEI scores and triglyceride concentrations. Similar to the results of linear regression models, DBI-TS scores were inversely associated with fasting serum triglyceride concentrations (OR = 0.965, 95% CI: 0.934 to 0.997, Pseudo R^2^ = 0.263; *p* = 0.032) in fully adjusted logistic model 2 (Supplementary Table S9), and the area under the curve (AUC) of this model was 75.7% (95% CI, 70.0% to 81.4%, Figure 1A).DBI-LBS scores were positively associated with fasting serum triglyceride concentrations (OR = 1.053, 95% CI: 1.011 to 1.097, Pseudo R^2^ = 0.270; *p* = 0.013) in fully adjusted logistic model 2 (Supplementary Table S9) and the AUC of this model was 76.0% (95% CI, 70.4% to 81.7%, Figure 1A). However, there was no significant association between CHEI scores and fasting serum triglyceride concentrations in fully adjusted logistic models (Supplementary Table S10). All *p*-values for the Hosmer-Lemeshow goodness of fit test were much more than 0.05. In addition, the calibration curves demonstrated that the apparent probabilities of triglyceride via DBI-TS and DBI-LBS scores were close to the ideal probability (Figure 2A,B).

In fully adjusted linear regression models (Model 2), DBI-TS and DQD scores were inversely associated with fasting serum TC:HDL-C ratio, and DBI-LBS scores were positively associated with fasting serum TC:HDL-C ratio (all *p* < 0.05) (Table 5). Further logistic regression analysis with ROC showed that DBI-TS scores were inversely associated with fasting serum TC:HDL-C ratio (OR = 0.948, 95% CI: 0.918 to 0.979, Pseudo R^2^ = 0.241; *p* = 0.001; AUC = 74.8%, CI: 68.9% to 80.6%, *p*-values for the Hosmer-Lemeshow test > 0.05) (Supplementary Table S9 and Figure 1). In contrast, DBI-LBS (OR = 1.069, 95% CI: 1.026 to 1.113, Pseudo R^2^ = 0.240, *p* = 0.001; AUC = 74.7%, 95% CI: 68.9% to 80.5%, *p*-values for the Hosmer-Lemeshow test > 0.05) and DQD (OR = 1.192, 95% CI: 1.079 to 1.316, Pseudo R^2^ = 0.250, *p* <0.001; AUC = 75.6%, 95% CI: 69.8% to 81.4%, *p*-values for the Hosmer-Lemeshow test > 0.05) scores were positively associated with fasting serum TC:HDL-C ratio (Supplementary Table S9 and Figure 1B). In addition, apparent calibration curves of DBI-TS, DBI-LBS, and DQD scores are consistent with the ideal lines (Figure 2E–G).

In fully adjusted linear regression models (Model 2), DBI-TS scores were positively associated with fasting serum HDL-C, and DBI-LBS scores were inversely associated with fasting serum concentrations of HDL-C (both *p* < 0.001) (Table 5). Similarly, further logistic regression analysis with ROC demonstrated that DBI-TS scores were positively associated with fasting serum concentrations of HDL-C (OR = 1.044, 95% CI: 1.010 to 1.079, Pseudo R^2^ = 0.283, *p* = 0.011, Supplementary Table S9) and the AUC of this model was 76.5% (95% CI: 70.9% to 82.1%, Figure 1C). In contrast, DBI-LBS scores were inversely associated with fasting serum concentrations of HDL-C (OR = 0.956, 95% CI: 0.918 to 0.996, Pseudo R^2^ = 0.275; *p* = 0.031, Supplementary Table S9) in fully adjusted logistic model 2 and the AUC of this model was 76.0% (95% CI: 70.4% to 81.7%, Figure 1C). All *p*-values for the Hosmer-Lemeshow test were much more than 0.05. Calibration curve of the models have shown that the apparent probabilities of fasting serum HDL-C via DBI-TS and DBI-LBS scores are consistent with the ideal probability (Figure 2C,D).

In fully adjusted linear regression models (Model 2), DBI-TS scores were positively associated with fasting serum concentrations of ApoA1, and DBI-LBS scores were inversely associated with fasting serum concentrations of ApoA1 (both *p* = 0.002) (Table 5). Consistent with these results, further logistic regression analyses demonstrated that DBI-TS (OR =1.052, 95% CI: 1.018 to 1.088, Pseudo R^2^ = 0.295, *p* = 0.002; AUC = 76.4%, 95% CI: 70.8% to 82.0%, *p*-values for the Hosmer-Lemeshow test > 0.05) and DBI-HBS (OR =1.073, 95% CI: 1.004 to 1.146, Pseudo R^2^ = 0.275, *p* = 0.038; AUC = 75.0%, 95% CI: 69.3% to 80.7%, *p*-values for the Hosmer-Lemeshow test > 0.05) scores were positively associated with fasting serum concentrations of ApoA1, and DBI-LBS scores were inversely associated with fasting serum concentrations of ApoA1 (OR = 0.948, 95% CI: 0.909 to 0.988, Pseudo R^2^ = 0.284, *p* = 0.011; AUC = 76.1%, 95% CI: 70.4% to 81.7%, *p*-values for the Hosmer-Lemeshow test > 0.05) (Supplementary Table S9 and Figure 1D). The apparent calibration curves of DBI-TS, DBI-LBS and DBI-HBS scores are close to the ideal lines (Figure 2G–I).

## 4. Discussion

Although much has been reported about dietary patterns and cardiometabolic health, studies directly investigate the associations between multiple a priori dietary indexes and cardiometabolic risk factors in the same group of participants are scarce, and available data for Chinese populations are strikingly limited, especially in hyperlipidemic patients. This missing information limits the attempts to update dietary guidelines for Chinese populations aimed at reducing cardiometabolic risk via dietary modifications. Our study was designed to address this research gap via assessing the associations between four frequently used a priori dietary indexes, including DBI-16, CHEI, MDS, and DASH scores, and cardiometabolic risk factors among Chinese hyperlipidemic patients in a cross-sectional setting. The unique aspect of our study is that we focused on a clinically relevant population, hyperlipidemic adults, who were at elevated risk for cardiometabolic disorders. To our knowledge, we provided the first comprehensive documentation of the associations between DBI-16, which emphasizes balanced dietary intake, and a broad range of cardiometabolic risk factors. The results of our work indicated that higher DBI-16, CHEI, and DASH scores were associated with more favorable lipid and lipoprotein profiles and/or glucose homeostasis biomarkers. There was no significant association between the four a prior dietary indexes and blood pressures.

DBI-16 was created based on eight food components from the most recent Dietary Guidelines for Chinese and the Chinese Food Pagoda and emphasized a balanced diet with adherence to these guidelines. The associations between DBI-16 scores and cardiometabolic risk factors differed by the four indicators of this index. As the DBI-TS scores increased, fasting serum triglyceride concentrations decreased and concentrations of HDL-C and ApoA1 increased. These results indicated that higher overall diet quality was associated with improved triglyceride and HDL-C concentrations. On the contrary, as the DBI-LBS scores increased, fasting serum triglyceride concentrations increased and concentrations of HDL-C and ApoA1 decreased. These data suggested that insufficient dietary intake resulted in unfavorable lipid and lipoprotein profiles, which may contribute to increased cardiometabolic risk. As a reflection for excessive dietary intake status, the DBI-HBS scores were positively associated with fasting serum glucose concentrations, indicating the potential role of excessive dietary intake in dysregulation of glucose homeostasis. In addition, our data demonstrated that better DQD scores were associated with more favorable values of a wide array of lipid and lipoprotein profiles, suggesting the importance of a balanced diet in the improvement of cardiometabolic health. Consistent with our findings, previous studies have found that low DBI scores are associated with unfavorable blood glucose and HDL-C concentrations [25] and higher prevalence of prediabetes [26] among Chinese adults. A study in European countries has also reported that in comparison to other behavioral risk factors, a balanced diet is a potential key factor to avoid cardiovascular disease-specific mortality [42]. Prior to our study, there are strikingly limited data on the relationships between balanced diet and cardiometabolic risk factors among hyperlipidemic Chinese adults. Lacking data in this area, randomized controlled-feeding trials among individuals with elevated cardiometabolic risk are needed to capture the alterations in cardiometabolic risk factors in response to a balanced diet compared to an unbalanced diet. We also observed differential patterns for associations between DBI scores and cardiometabolic risk factors in female and male participants, indicating that sex-specific recommendations should be considered for choosing dietary patterns to improve cardiometabolic health.

We observed a negative association between CHEI scores and fasting serum triglyceride concentrations. This result is consistent with previous studies [43,44]. This finding may be partially attributed to the relatively lower intakes of red and processed meats and saturated fatty acids and higher intakes of legume and legume products in participants with higher CHEI scores. High intakes of red and processed meats and saturated fatty acids have been reported to be associated with increased prevalence of hypertriglyceridemia [45,46], and substitution legumes for red meats results in significant reduction in triglyceride concentrations in overweight type 2 diabetic patients [47]. Compared to DBI-16, CHEI scores were not associated with other lipid and lipoprotein profiles or glucose homeostasis biomarkers, indicating that CHEI may not be suitable for comprehensive evaluations of the relationships between diet quality and cardiometabolic risk factors. Of note, CHEI is one-sided and has only one overall score to reflect whether an individual’s overall dietary intakes meet recommended requirements of dietary guidelines. We cannot rule out the possibility that excessive food and nutrient intakes obscured potential associations between associations between diet quality and cardiometabolic risk factors.

Our study provided the first documentation that the MDS scores were only associated with fasting HbA1c concentrations in study participants in models adjusted for age, sex and BMI, and the association was no longer significant in fully adjusted models. Possible explanation of this null finding may be attributed to the extremely low MDS scores in these participants, indicating low adherence to the traditional Mediterranean diet. Due to the differences in dietary, social and cultural background between China and the Mediterranean regions, the traditional Mediterranean diet may not be appropriate for instructing dietary intakes among Chinese populations.

In our study, the DASH scores were significantly associated with decreased concentrations of fasting blood glucose. The potential underlying mechanisms responsible for the association may be attributed, in part, to emphasis in higher intakes of fruits, vegetables, low-fat dairy products, whole grains, nuts and legumes and lower intakes of total and saturated fatty acids in the DASH dietary pattern [22]. The intakes of fruits, vegetables, low-fat dairy products, whole grains, and nuts and legumes individually or collectively contribute to blood glucose homeostasis [48,49,50]. In addition, higher adherence to the DASH dietary pattern has been reported to be associated with decreased incidence of type 2 diabetes [51]. No association between the DASH score and blood pressures was observed in our study. Although this null finding was somewhat unexpected, it is consistent with a previous randomized controlled trial in Hong Kong, which has reported that nutrition counselling with the DASH diet combined with physician’s usual care for 12 months has no significantly different effect on systolic and diastolic blood pressures compared with physician’s usual care alone [52]. However, a cross-sectional study has demonstrated inverse associations between DASH scores and prevalence of hypertension in Chinese adults [53], while another multi-ethnic group study including Chinese individuals has reported opposite conclusions [54]. Collectively, it is still controversial as to whether the DASH diet could be used to assist Chinese populations in the prevention of hypertension as well as other cardiometabolic risks; hence, further research is required on this topic.

There are several strengths in this study. Four a priori (hypothesis-driven) dietary indexes were used, which captured the overall quality and complexity of diet and possible interplay among foods and nutrients. The target group consisted of hyperlipidemic patients who were at high-risk for cardiometabolic disorders and were most likely to benefit from dietary modifications for optimizing cardiometabolic risk factors. A broader range of dietary indexes was assessed than previously reported. A limitation of this study was that with the observational nature of this study, causality could not be interpreted and potential mechanisms underlying the associations between a priori dietary indexes and cardiometabolic risk factors were not investigated.

## 5. Conclusions

In conclusion, better DBI-16, CHEI, and DASH scores were associated with more favorable lipid and lipoprotein profiles and/or glucose homeostasis biomarkers among Chinese hyperlipidemic participants in this cross-sectional study. There was no significant association between the four a prior dietary indexes and blood pressures. Among four a priori dietary indexes assessed, DBI-16 was more suitable for a comprehensive evaluation of the overall diet quality and balance for optimizing cardiometabolic health among hyperlipidemic individuals. In particular, better DQD scores were associated with more favorable fasting serum cholesterol and lipoprotein profiles. These findings add new information to the current literature, suggesting that maintaining a balanced diet could be incorporated in current dietary guidelines for the Chinese population, aimed at reducing cardiometabolic risk.

## Figures and Tables

**Table 1:** Characteristics of 269 Chinese participants with hyperlipidemia according to gender ^1,2^.

Variables	All (*n* = 269)	Female (*n* = 202)	Male (*n* = 67)	*p* Values
Age, year	58 ± 8	58 ± 7	57 ± 9	0.219
Body weight, kg	60.9 ± 10.9	57.8 ± 9.1	70.2 ± 10.7	<0.001
Height, m	158.6 ± 8.2	155.6 ± 5.8	167.8 ± 7.3	<0.001
BMI, kg/m^2^	24.1 ± 3.2	23.9 ± 3.2	24.9 ± 3.1	0.024
Underweight (BMI < 18.5)	12 (4.5%)	2 (3.0%)	10 (5.0%)	0.563
Normal weight (18.5 ≤ BMI ≤ 23.9)	120 (44.6%)	26 (38.8%)	94 (46.5%)	
Overweight (24.0 ≤ BMI ≤ 27.9)	106 (39.4%)	30 (44.8%)	76 (37.6%)	
Obese (BMI ≥ 28)	31 (11.5%)	9 (13.4%)	22 (10.9%)	
Waist circumference, cm	85.2 ± 9.9	83.5 ± 10.0	90.2 ± 7.8	<0.001
Central obesity				0.642
Yes	119 (44.2%)	91 (45.0%)	28 (41.8%)	
No	150 (55.8%)	111 (55.0%)	39 (58.2%)	
Physical activity status (MET-h/week)	94.9 ± 70.3	100.0 ± 78.2	79.6 ± 33.5	<0.001
Marital status				0.182
Married	249 (92.6%)	184 (91.1%)	65 (97.0%)	
Other	20 (7.4%)	18 (8.9%)	2 (3.0%)	
Education				0.006
Primary school	8 (3.0%)	8 (4.0%)	0 (0%)	
Junior high school	37 (13.8%)	28 (13.9%)	9 (13.4%)	
High school/secondary school	107 (39.8%)	83 (41.1%)	24 (35.8%)	
College	70 (26.0%)	57 (28.2%)	13 (19.4%)	
Bachelor’s degree or postgrad	47 (17.5%)	26 (12.9%)	21 (31.3%)	
Smoking status				<0.001
Yes	18 (6.7%)	0 (0%)	18 (26.9%)	
No	251 (93.3%)	202 (100%)	49 (73.1%)	
Employment				<0.001
Full-time	70 (26.0%)	36 (17.8%)	34 (50.7%)	
Part-time	10 (3.7%)	9 (4.5%)	1 (1.5%)	
Other	189 (70.3%)	157 (77.8%)	32 (47.8%)	

^1^ Data are presented as mean ± SD or *n* (%). MET, metabolic equivalent tasks. ^2^ BMI categories were based on criteria for Chinese adults: underweight defined as BMI < 18.5 kg/m^2^; normal weight defined as 18.5 ≤ BMI ≤ 23.9 kg/m^2^; overweight as 24 ≤ BMI ≤ 27.9 kg/m^2^; obese defined as BMI ≥ 28 kg/m^2^. Central obesity was defined as waist circumference ≥ 90 cm for men and ≥ 85 cm for women based on criteria for Chinese adults. Differences in characteristics between female and male participants were compared using the 2-sample *t* test for continuous variables or chi-square for categorical variables.

**Table 2:** Cardiometabolic risk factor levels of 269 Chinese participants with hyperlipidemia according to gender ^1,2^.

Cardiometabolic Risk Factors	All (*n* = 269)	Female (*n* = 202)	Male (*n* = 67)	*p* Values
Lipid and lipoprotein profiles				
Triglyceride, mmol/L	2.0 ± 1.4	1.9 ± 1.3	2.5 ± 1.7	0.020
TC, mmol/L	6.2 ± 1.0	6.3 ± 1.0	5.8 ± 1.0	<0.001
LDL-C, mmol/L	4.2 ± 1.0	4.3 ± 1.0	3.9 ± 1.0	0.004
HDL-C, mmol/L	1.5 ± 0.4	1.6 ± 0.4	1.3 ± 0.4	<0.001
TC:HDL-C ratio	4.4 ± 1.2	4.2 ± 1.2	4.9 ± 1.2	<0.001
LDL-C:HDL-C ratio	3.0 ± 0.9	2.9 ± 0.9	3.2 ± 0.8	0.005
ApoA1, g/L	1.5 ± 0.3	1.5 ± 0.3	1.3 ± 0.2	<0.001
ApoB, g/L	1.3 ± 0.3	1.3 ± 0.3	1.3 ± 0.2	0.176
ApoA1:ApoB ratio	1.2 ± 0.4	1.2 ± 0.4	1.1 ± 0.3	0.004
Glucose homeostasis biomarkers				
Glucose, mmol/L	5.4 ± 1.1	5.4 ± 1.1	5.4 ± 1.1	0.938
HbA1c, %	5.8 ± 0.7	5.8 ± 0.7	5.7 ± 0.7	0.765
Blood pressure				
Systolic blood pressure, mm Hg	115.1 ± 16.7	113.2 ± 16.6	120.6 ± 16.1	0.002
Diastolic blood pressure, mm Hg	73.2 ± 10.2	71.6 ± 9.8	78.0 ± 9.9	<0.001

^1^ Data are presented as mean ± SD. Apo, apolipoprotein; HbA1c, glycated hemoglobin A1c; HDL-c, high density lipoprotein; LDL-c, low density lipoprotein cholesterol; TC, total cholesterol. ^2^ Differences in cardiometabolic risk factor levels between female and male participants were compared using 2-sample *t* test.

**Table 3:** Daily dietary and macronutrient intake of 269 Chinese participants with hyperlipidemia according to gender ^1,2^.

Food Groups and Macronutrients	All (*n* = 269)	Female (*n* = 202)	Male (*n* = 67)	*p* Values
Food groups, g/day				
Total fruits	201.3 ± 164.3	204.6 ± 164.2	191.4 ± 165.4	0.569
Total vegetables	393.2 ± 249.3	407.6 ± 258.8	349.8 ± 214.1	0.100
Dark green/orange vegetables	307.3 ± 206.4	317.4 ± 212.1	277.1 ± 186.2	0.167
Total grains	392.9 ± 143.7	377.3 ± 141.0	439.7 ± 142.5	0.002
Red and processed meats	75.4 ± 50.0	74.7 ± 48.0	77.3 ± 55.9	0.712
Fish, shellfish and mollusk	42.8 ± 43.2	41.9 ± 43.5	45.6 ± 42.9	0.553
Eggs	38.9 ± 24.5	40.8 ± 26.0	33.1 ± 18.4	0.025
Dairy and dairy products	130.8 ± 113.0	139.1 ± 112.6	105.8 ± 111.6	0.036
Soybean and soybean products	17.7 ± 20.7	18.3 ± 22.2	15.9 ± 15.1	0.415
Alcohol	1.7 ± 11.3	0.2 ± 0.8	6.2 ± 22.0	0.029
Energy and macronutrients				
Energy, kcal	1643.4 ± 653.2	1551.8 ± 574.7	1919.4 ± 789.6	<0.001
Carbohydrates, % E	43.2 ± 8.9	42.7 ± 8.9	45.0 ± 8.5	0.063
Soluble fibers, g/d	12.5 ± 4.4	12.9 ± 4.4	11.5 ± 4.3	0.031
Fats, % E	41.1 ± 9.2	41.6 ± 9.3	39.7 ± 8.7	0.145
PUFAs, % E	11.9 ± 4.6	12.1 ± 4.7	11.0 ± 4.1	0.097
MUFAs, % E	15.2 ± 4.2	15.3 ± 4.3	14.6 ± 3.9	0.216
SFAs, % E	10.1 ± 2.3	10.2 ± 2.3	9.7 ± 2.3	0.091
Proteins, % E	17.3 ± 3.6	17.5 ± 3.7	16.8 ± 3.4	0.234

^1^ Data are presented as mean ± SD. MUFA: monounsaturated fatty acids; PUFA: polyunsaturated fatty acids; SFA: saturated fatty acids. ^2^ Intakes of food groups and soluble fiber were adjusted as grams for 1643 kcal energy (average daily energy intake). Differences in food and macronutrient intake between female and male participants were compared using 2-sample *t* test.

**Table 4:** Distribution of a priori dietary index scores among 269 Chinese participants with hyperlipidemia according to gender ^1,2^.

Dietary Indexes and Scoring Systems	All (*n* = 269)	Female (*n* = 202)	Male (*n* = 67)	*p* Values
DBI-16				
DBI-TS				0.928
Excessive dietary intake (>0)	161 (59.9%)	120 (59.4%)	41 (61.2%)	
Excellent dietary intake (0)	7 (2.6%)	5 (2.5%)	2 (3.0%)	
Insufficient dietary intake (<0)	101 (37.5%)	77 (38.1%)	24 (35.8%)	
DBI-LBS				0.458
Excellent dietary intake (0)	0 (0%)	0 (0%)	0 (0%)	
Good dietary intake (1–14)	135 (50.2%)	105 (52.0%)	30 (44.8%)	
Acceptable dietary intake (15–29)	131 (48.7%)	95 (47.0%)	36 (53.7%)	
Poor dietary intake (29–43)	3 (1.1%)	2 (1.0%)	1 (1.5%)	
Worst dietary intake (>43)	0 (0%)	0 (0%)	0 (0%)	
DBI-HBS				0.385
Excellent dietary intake (0)	0 (0%)	0 (0%)	0 (0%)	
Good dietary intake (1–9)	25 (9.3%)	20 (9.9%)	5 (7.5%)	
Acceptable dietary intake (10–18)	116 (43.1%)	85 (42.1%)	31 (46.3%)	
Poor dietary intake (19–27)	127 (47.2%)	97 (48.0%)	30 (44.8%)	
Worst dietary intake (>27)	1 (0.4%)	0 (0%)	1 (1.5%)	
DQD				1.000
Excellent dietary intake (0)	0 (0%)	0 (0%)	0 (0%)	
Good dietary intake (1–19)	262 (97.4%)	197 (97.5%)	65 (97.0%)	
Low imbalanced diet (20–38)	7 (2.6%)	5 (2.5%)	2 (3.0%)	
Moderate imbalanced diet (39–57)	0 (0%)	0 (0%)	0 (0%)	
High imbalanced diet (>57)	0 (0%)	0 (0%)	0 (0%)	
CHEI				0.902
Unqualified (<60)	31 (11.5%)	23 (11.4%)	8 (11.9%)	
Qualified (≥60)	238 (88.5%)	179 (88.6%)	59 (88.1%)	
MDS				0.185
Low (≤6)	200 (74.3%)	151 (74.8%)	49 (73.1%)	
Low–medium (7)	45 (16.7%)	36 (17.8%)	9 (13.4%)	
Medium–high (8)	21 (7.8%)	12 (5.9%)	9 (13.4%)	
High (9–14)	3 (1.1%)	3 (1.5%)	0 (0%)	
DASH score				0.211
Lowest (0–27)	26 (9.7%)	19 (9.4%)	7 (10.4%)	
Medium (28–53)	234 (87.0%)	174 (86.1%)	60 (89.6%)	
Highest (54–80)	9 (3.3%)	9 (4.5%)	0 (0%)	

^1^ Data are presented as *n* (%). CHEI, Chinese Healthy Eating Index; DASH, dietary approaches to stop hypertension; DBI, Diet Balance Index; DBI-HBS, diet balance index-high bound score; DBI-LBS, Diet Balance Index-low bound scores; DBI-TS, Diet Balance Index-total score; DQD, diet quality distance; MDS, Mediterranean Diet Score. ^2^ Differences in a priori dietary indexes between female and male participants were compared using chi-square.

**Table 5:** Associations between DBI-16 scores and cardiometabolic risk factors among 269 Chinese participants with hyperlipidemia ^1,2^.

Cardiometabolic Risk Factors	DBI-TS		DBI-LBS		DBI-HBS		DQD	
	β Coefficient (95% CI)	R^2^	β Coefficient (95% CI)	R^2^	β Coefficient (95% CI)	R^2^	β Coefficient (95% CI)	R^2^
Lipid and lipoprotein profiles								
Triglyceride								
Model 1	−0.021 (−0.040, −0.002) *	11.5%	0.028 (0.005, 0.052) *	11.8%	−0.010 (−0.044, 0.024)	10.1%	0.009 (−0.037, 0.055)	10.0%
Model 2	−0.024 (−0.043, −0.005) *	13.3%	0.032 (0.008, 0.055) *	13.5%	−0.017 (−0.055, 0.021)	11.6%	0.011 (−0.044, 0.065)	11.4%
TC								
Model 1	0.011 (−0.002, 0.024)	12.3%	−0.007 (−0.023, 0.009)	11.7%	0.022 (−0.002, 0.045)	12.5%	0.034 (0.003, 0.066) *	12.9%
Model 2	0.011 (−0.002, 0.024)	12.7%	−0.008 (−0.025, 0.008)	12.2%	0.022 (−0.004, 0.048)	12.8%	0.040 (0.003, 0.078) *	13.3%
LDL-C								
Model 1	0.010 (−0.004, 0.024)	10.2%	−0.006 (−0.024, 0.011)	9.7%	0.019 (−0.006, 0.044)	10.3%	0.040 (0.006, 0.074) *	11.4%
Model 2	0.011 (−0.003, 0.025)	10.7%	−0.008 (−0.026,0.010)	10.2%	0.023 (−0.005,0.051)	10.8%	0.052 (0.012, 0.092) *	12.1%
HDL-C								
Model 1	0.009 (0.004, 0.015) *	26.9%	−0.012 (−0.019, −0.005) *	27.0%	0.006 (−0.004, 0.015)	24.1%	−0.013 (−0.027, 0) *	24.8%
Model 2	0.010 (0.004, 0.015) *	28.8%	−0.013 (−0.020, −0.006) *	29.1%	0.007 (−0.004, 0.018)	25.7%	−0.020 (−0.036, −0.005) *	27.1%
TC:HDL-C ratio								
Model 1	−0.021 (−0.037, −0.005) *	14.5%	0.031 (0.011, 0.051) *	15.4%	−0.003 (−0.032, 0.026)	12.4%	0.053 (0.014, 0.092) *	14.7%
Model 2	−0.023 (−0.039, −0.007) *	16.4%	0.034 (0.014, 0.054) *	17.1%	−0.010 (−0.042, 0.023)	13.9%	0.068 (0.023, 0.114) *	16.5%
LDL-C:HDL-C ratio								
Model 1	−0.010 (−0.022, 0.002)	9.3%	0.019 (0.004, 0.034) *	10.5%	0.006 (−0.016, 0.027)	8.5%	0.053 (0.024, 0.082) *	12.7%
Model 2	−0.011 (−0.023, 0.001)	10.1%	0.020 (0.005, 0.035) *	11.2%	0.005 (−0.019, 0.029)	9.1%	0.071 (0.037, 0.104) *	14.5%
ApoA1								
Model 1	0.005 (0.002, 0.008) *	23.4%	−0.006 (−0.010, −0.002) *	23.0%	0.004 (−0.002, 0.010)	21.5%	−0.007 (−0.015, 0.002)	21.7%
Model 2	0.005 (0.002, 0.008) *	25.6%	−0.006 (−0.011, −0.002) *	25.4%	0.005 (−0.002, 0.011)	23.4%	−0.011 (−0.021,−0.002) *	24.3%
ApoB								
Model 1	0.001 (−0.002, 0.005)	5.9%	0.001 (−0.004, 0.005)	5.8%	0.005 (−0.001, 0.012)	6.7%	0.015 (0.006, 0.023) *	9.7%
Model 2	0.001 (−0.002, 0.005)	6.3%	0 (−0.004, 0.005)	6.2%	−0.005 (−0.002, 0.012)	6.8%	0.017 (0.007, 0.027) *	10.1%
ApoA1:ApoB ratio								
Model 1	0.001 (−0.004, 0.006)	7.9%	−0.003 (−0.009, 0.003)	8.2%	−0.003 (−0.012, 0.005)	8.0%	−0.021 (−0.033, −0.010) *	12.1%
Model 2	0.001 (−0.003, 0.006)	8.7%	−0.003 (−0.009, 0.003)	9.0%	−0.003 (−0.012, 0.007)	8.7%	−0.027 (−0.041, −0.014) *	13.6%
Glucose homeostasis biomarkers								
Glucose								
Model 1	0.009 (−0.006, 0.024)	6.0%	0.001 (−0.017, 0.020)	5.5%	0.032 (0.005, 0.058) *	7.4%	0.030 (−0.006, 0.066)	6.4%
Model 2	0.008 (−0.007, 0.023)	7.0%	0 (−0.019, 0.019)	6.6%	0.032 (0.003, 0.062) *	8.2%	0.030 (−0.012, 0.073)	7.3%
HbA1c								
Model 1	0.005 (−0.005, 0.015)	6.0%	0.001 (−0.011, 0.013)	5.6%	0.018 (0.001, 0.036) *	7.1%	0.024 (0, 0.047) *	6.9%
Model 2	0.005 (−0.005, 0.014)	8.2%	0 (−0.013, 0.012)	7.9%	0.017 (−0.002, 0.036)	8.9%	0.023 (−0.005, 0.050)	8.8%
Blood pressures								
Systolic blood pressure								
Model 1	−0.039 (−0.252, 0.174)	17.8%	0.012 (−0.256, 0.279)	17.7%	−0.103 (−0.486, 0.280)	17.8%	−0.380 (−0.898, 0.138)	18.4%
Model 2	−0.027 (−0.242, 0.188)	18.7%	0.059 (−0.213, 0.331)	18.8%	0.037 (−0.391, 0.465)	18.7%	−0.206 (−0.820, 0.408)	18.9%
Diastolic blood pressure								
Model 1	0 (−0.128, 0.129)	19.2%	−0.080 (−0.241, 0.080)	19.5%	−0.164 (−0.394, 0.066)	19.7%	−0.234 (−0.546, 0.078)	19.8%
Model 2	0.012 (−0.117, 0.140)	21.2%	−0.045 (−0.207, 0.118)	21.3%	−0.065 (−0.320, 0.191)	21.3%	−0.060 (−0.427, 0.308)	21.3%

^1^ Data are presented as β coefficients (95% CI) per 1 SD of the DBI-16 score. Apo, Apolipoprotein; DBI, Diet Balance Index; DBI-HBS, diet balance index-high bound score; DBI-LBS, Diet Balance Index-low bound scores; DBI-TS, Diet Balance Index-total score; DQD, diet quality distance; HbA1c, glycated hemoglobin A1c; HDL-C, high density lipoprotein; LDL-C, low density lipoprotein cholesterol; TC, total cholesterol. ^2^ Associations between the DBI-16 scores and cardiometabolic risk factors were analyzed using multivariable linear regression models. In model 1, the data were adjusted for potential confounders, including age, sex, BMI. Model 2 included model 1 plus additional sociodemographic and lifestyle confounders, including cigarette smoking, education status, physical activity and total energy intake. * *p* < 0.05.

**Table 6:** Associations between CHEI, MDS, and DASH scores and cardiometabolic risk factors among 269 Chinese participants with hyperlipidemia ^1,2^.

Cardiometabolic Risk Factors	CHEI		MDS		DASH Scores	
	β Coefficient (95% CI)	R^2^	β Coefficient (95% CI)	R^2^	β Coefficient (95% CI)	R^2^
Lipid and lipoprotein profiles						
Triglyceride						
Model 1	−0.015 (−0.032, 0.002)	11.0%	−0.009 (−0.012, 0.135)	10.0%	−0.010 (−0.031, 0.010)	10.3%
Model 2	−0.018 (−0.035, −0.001) *	12.8%	0.002 (−0.148, 0.151)	11.3%	−0.011 (−0.032, 0.010)	11.7%
TC						
Model 1	0.003 (−0.009, 0.014)	11.5%	0.033 (−0.066, 0.131)	11.6%	0.005 (−0.010, 0.019)	11.6%
Model 2	0.003 (−0.009, 0.015)	11.9%	0.051 (−0.052, 0.155)	12.2%	0.006 (−0.009, 0.020)	12.1%
LDL-C						
Model 1	0.004 (−0.008, 0.017)	9.7%	0.017 (−0.088, 0.123)	9.6%	0.003 (−0.012, 0.019)	9.6%
Model 2	0.005 (−0.007, 0.018)	10.2%	0.025 (−0.086, 0.136)	10.0%	0.004 (−0.012, 0.019)	10.0%
HDL-C						
Model 1	0.003 (−0.002, 0.008)	24.2%	0.015 (−0.026, 0.057)	23.9%	0.003 (−0.003, 0.009)	24.1%
Model 2	0.004 (−0.001, 0.009)	26.0%	0.018 (−0.025, 0.061)	25.5%	0.004 (−0.002, 0.010)	25.8%
TC:HDL-C ratio						
Model 1	−0.009 (−0.024, 0.005)	12.9%	−0.024 (−0.146, 0.099)	12.5%	−0.013 (−0.030, 0.005)	13.0%
Model 2	−0.012 (−0.026, 0.003)	14.6%	−0.007 (−0.135, 0.120)	13.8%	−0.013 (−0.030, 0.005)	14.4%
LDL-C:HDL-C ratio						
Model 1	−0.003 (−0.014, 0.008)	8.5%	−0.028 (−0.118, 0.063)	8.5%	−0.007 (−0.020, 0.006)	8.7%
Model 2	−0.004 (−0.015, 0.007)	9.2%	−0.023 (−0.118, 0.072)	9.1%	−0.007 (−0.020, 0.006)	9.4%
ApoA1						
Model 1	0.001 (−0.002, 0.004)	21.0%	0.009 (−0.016, 0.034)	21.1%	0.002 (−0.002, 0.006)	21.2%
Model 2	0.001 (−0.002, 0.004)	23.0%	0.012 (−0.014, 0.038)	23.1%	0.002 (−0.001, 0.006)	23.3%
ApoB						
Model 1	0.001 (−0.003, 0.004)	5.8%	0.002 (−0.027, 0.027)	5.8%	0 (−0.004, 0.004)	5.8%
Model 2	0.001 (−0.003, 0.004)	6.2%	0.004 (−0.027, 0.032)	6.2%	0 (−0.004, 0.004)	6.2%
ApoA1:ApoB ratio						
Model 1	−0.002 (−0.006, 0.003)	8.0%	0.006 (−0.030, 0.043)	7.9%	0 (−0.005, 0.006)	7.8%
Model 2	−0.001 (−0.006, 0.003)	8.7%	0.003 (−0.034, 0.041)	8.6%	0.001 (−0.005, 0.006)	8.6%
Glucose homeostasis biomarkers						
Glucose						
Model 1	−0.002 (−0.016, 0.011)	5.5%	−0.092 (−0.201, 0.020)	6.4%	−0.018 (−0.034, −0.002) *	7.1%
Model 2	−0.002 (−0.016, 0.011)	6.6%	−0.073 (−0.190, 0.044)	7.1%	−0.017 (−0.033, 0) *	8.0%
HbA1c						
Model 1	−0.002 (−0.011, 0.007)	5.7%	−0.088 (−0.160, −0.015) *	7.5%	−0.010 (−0.020, 0.001)	6.7%
Model 2	−0.001 (−0.010, 0.007)	7.9%	−0.073 (−0.149, 0.003)	9.1%	−0.009 (−0.019, 0.002)	8.7%
Blood pressures						
Systolic blood pressure						
Model 1	−0.071 (−0.263, 0.122)	17.9%	0.189 (−1.428, 1.806)	17.8%	−0.148 (−0.382, 0.087)	18.2%
Model 2	−0.062 (−0.256, 0.132)	18.8%	−0.132 (−1.822, 1.558)	18.7%	−0.153 (−0.387, 0.082)	19.2%
Diastolic blood pressure						
Model 1	0.023 (−0.093, 0.139)	19.2%	0.377 (−0.596, 1.349)	19.3%	0.043 (−0.098, 0.185)	19.3%
Model 2	−0.030 (−0.087, 0.146)	21.3%	0.065 (−0.945, 1.076)	21.2%	0.039 (−0.102, 0.179)	21.3%

^1^ Data are presented as β coefficients (95% CI) per 1 SD of the CHEI, MDS, or DASH score. Apo, Apolipoprotein; CHEI, Chinese Healthy Eating Index; DASH, dietary approaches to stop hypertension; HbA1c, glycated hemoglobin A1c; HDL-C, high density lipoprotein; LDL-C, low density lipoprotein cholesterol; MDS, Mediterranean Diet Score; TC, total cholesterol. ^2^ Associations between the CHEI, MDS, DASH scores and cardiometabolic risk factors were analyzed using multivariable linear regression models. In model 1, the data were adjusted for potential confounders, including age, sex, BMI. Model 2 included model 1 plus additional sociodemographic and lifestyle confounders, including cigarette smoking, education status, physical activity and total energy intake. * *p* < 0.05.

## Data Availability

Data described in the manuscript, code book, and analytic code will be made available upon reasonable request.

## Supplementary Materials

The following are available online at https://www.mdpi.com/article/10.3390/nu13072179/s1, Figure S1: Flow diagram of recruitment and screening for participants with hyperlipidemia, Table S1: DBI-16 components and standard for scoring, Table S2: CHEI components and standard for scoring, Table S3: MDS components and standard for scoring, Table S4: DASH score components and standard for scoring, Table S5: Associations between DBI-16 scores and cardiometabolic risk factors among 202 female participants with hyperlipidemia, Table S6: Associations between DBI-16 scores and cardiometabolic risk factors among 67 male participants with hyperlipidemia, Table S7: Associations between CHEI, MDS, and DASH scores and cardiometabolic risk factors among 202 female participants with hyperlipidemia, Table S8: Associations between CHEI, MDS, and DASH scores and cardiometabolic risk factors among 67 male participants with hyperlipidemia, Table S9: Associations between DBI-16 scores and cardiometabolic risk factors among 269 Chinese participants with hyperlipidemia Table S10: Associations between CHEI, MDS, and DASH scores and cardiometabolic risk factors among 269 Chinese participants with hyperlipidemia.
