# Supplementary material for: Associations between Adherence to Four A Priori Dietary Indexes and Cardiometabolic Risk Factors among Hyperlipidemic Patients
Source: Nutrients. 2021 Jun 24;13(7):2179. doi: 10.3390/nu13072179 (PMC8308401; doi:10.3390/nu13072179)
Supplement: Supplementary file 1 [file nutrients-13-02179-s001.zip › Supplementary Tables20210617.pdf]

Supplementary Table S1. DBI-16 components and standard for scoring[1].

Component	Score	Subgroup	Score	Intake range by energy intake level										
				1000 kcal	1200 kcal	1400 kcal	1600 kcal	1800 kcal	2000 kcal	2200 kcal	2400 kcal	2600 kcal	2800 kcal	3000 kcal
C1-Cereal	(-12)-12	Cereal	(-12)-12	0g=-12<br>75-95g=0<br>>170g=12	<15g=-12<br>90-110g=0<br>>185g=12	0g=-12<br>125-175g=0<br>>250g=12	<10g=-12<br>175-225g=0<br>>390g=12	<35g=-12<br>200-250g=0<br>>415g=12	<5g=-12<br>225-275g=0<br>>495g=12	<30g=-12<br>250-300g=0<br>>520g=12	0g=-12<br>275-325g=0<br>>600g=12	<50g=-12<br>325-375g=0<br>>650g=12	<75g=-12<br>350-400g=0<br>>675g=12	<100g=-12<br>375-425g=0<br>>700g=12
C2-vegetable and fruit	(-12)-0	Vegetable	(-6)-0	≥200g=0<br>160-199g=-1<br>Score decreased 1 with intake amount decreased 40g<br>0g=-6	≥250g=0<br>200-249g=-1<br>Score decreased 1 with intake amount decreased 50g<br>0g=-6	≥3000g=0<br>240-299g=-1<br>Score decreased 1 with intake amount decreased 60g<br>0g=-6		≥400g=0<br>320-399g=-1<br>Score decreased 1 with intake amount decreased 80g<br>0g=-6	≥450g=0<br>360-449g=-1<br>Score decreased 1 with intake amount decreased 90g<br>0g=-6		≥500g=0<br>400-499g=-1<br>Score decreased 1 with intake amount decreased 1000g<br>0g=-6			≥600g=0<br>480-599g=-1<br>Score decreased 1 with intake amount decreased 1200g<br>0g=-6
		Fruit	(-6)-0	⩾150g=0; 120-149g=-1<br>Score decreased 1 with intake amount decreased 30g<br>0g=-6			⩾2000g=0; 160-199g=-1<br>Score decreased 1 with intake amount decreased 40g<br>0g=-6		⩾300g=0; 240-299g=-1<br>Score decreased 1 with intake amount decreased 60g<br>0g=-6		⩾350g=0; 280-349g=-1<br>Score decreased 1 with intake amount decreased 70g<br>0g=-6		⩾400g=0; 320-399g=-1<br>Score decreased 1 with intake amount decreased 80g<br>0g=-6	
C3-Milk and dairy products Soybean and soybean products	(-12)-0	Dairy	(-6)-0	≥500g=0<br>Score decreased 1 with intake amount decreased 100g<br>0g=-6		≥350g=0<br>Score decreased 1 with intake amount decreased 70g<br>0g=-6	≥300g=0<br>Score decreased 1 with intake amount decreased 60g<br>0g=-6							
		Soybean	(-6)-0	⩾5g=0	⩾15g=0	⩾25g=0								

				Score decreased 1 with intake amount decreased 1g<br>0g=-6	Score decreased 1 with intake amount decreased 3g<br>0g=-6					Score decreased 1 with intake amount decreased 5g<br>0g=-6		
C4-Animal food	(-12)-8	Red meat and products, Poultry and game	(-4)-4	0g=-3<br>1-5g=-2<br>6-10g=-1<br>11-20g=0<br>21-25g=1<br>26-30g=2<br>31-35g=3<br>>35g=4	0g=-4<br>1-5g=-3<br>6-10g=-2<br>11-15g=-1<br>16-35g=0<br>36-40g=1<br>41-45g=2<br>46-50g=3<br>>50g =4	0g=-4<br>1-10g=-3<br>11-20g=-2<br>21-30g=-1<br>31-50g=0<br>51-60g=1<br>61-70g=2<br>71-80g=-3<br>>80g =4		0g=-4<br>1-15g=-3<br>16-30g=-2<br>31-45g=-1<br>46-55g=0<br>56-70g=1<br>71-85g=2<br>85-100g=3<br>>100g =4		0g=-4<br>1-20g=-3<br>21-40g=-2<br>41-60g=-3<br>61-90g=0<br>91-110g=1<br>111-130g=2<br>131-150g=3<br>>150g=4	0g=-4<br>1-25g=-3<br>26-50g=-2<br>51-75g=-1<br>76-125g=0<br>126-150g=1<br>151-175g=2<br>176-200g=3<br>>200g=4	
		Fish and Shrimp	(-4)-0	0g=-4<br>1-4g=-3<br>5-9g=-2<br>10-14g=-1<br>⩾15g=0	<5g=-4<br>5-9g=-3<br>10-14g=-2<br>15-19g=-1<br>⩾20g=0	<10g=-4<br>10-19g=-3<br>20-29g=-2<br>30-39g=-1<br>⩾40g=0		<5g=-4<br>5-19g=-3<br>20-34g=-2<br>35-49g=-1<br>⩾50g=0		0g=-4<br>1-24g=-3<br>25-49g=-2<br>50~74g=-1<br>⩾75g=0	<25g=-4<br>25-49g=-3<br>50-74g=-2<br>75-99g=-1<br>⩾100g=0	<50g=-4<br>50-74g=-3<br>75-99g=-2<br>100-124g=-1<br>⩾125g=0
		Egg	(-4)-4	0g=-4<br>1-5g=-3<br>6-10g=-2<br>11-15g=-1<br>16-25g=0<br>26-30g=1	<5g=-4<br>6-10g=-3<br>11-15g=-2<br>16-20g=-1<br>21-30g=0<br>31-35g=1		0g=-4<br>1-10g=-3<br>11-20g=-2<br>21-30g=-1<br>31-50g=0<br>51-60g=1		0g=-4<br>1-15g=-3<br>16-30g=-2<br>31-45g=-1<br>46-55g=0<br>56-70g=1			

				31-35g=2<br>36-40g=3<br>>40g=4	36-40g=2<br>41-45g=3<br>>45g =4		61-70g=2<br>71-80g=-3<br>>80g =4	71-85g=2<br>85-100g=3<br>>100g =4		
C5-Empty energy food	0-12	Cooking oil	0-6	≤20g=0<br>21-25g=1<br>>45g=6	≤25g=0<br>26-30g=1<br>>50g=6				≤30g=0<br>31-35g=1<br>>55g=6	≤35g=0<br>36-40g=1<br>>60g=6
		Alcoholic beverage	0-6	Male: ≤ 25g=0; 26-40g=1; score increased 1 with intake amount increased 15g; >100g=6<br>(25g alcohol=750ml beer or 250ml wine or 75g liquor 38°or 50g liquor > 38°)<br>Female: ≤15g=0; 16-25g=1; score increased 1 with intake amount increased 10g; >65g=6<br>(15g alcohol=450ml beer or 150ml wine or 50g liquor 38° or 30g liquor > 38°)						
C6-Condiments	0-12	Addible sugar	0-6	≤25g=0; 26g=1; score increased 1 with intake amount increased 5g; >50g=6						
		Salt	0-6	<2g=0<br>2-3g=1<br>Score increased 1 with intake amount increased 2g<br>>12g=6	<3g=0<br>3-4g=1<br>score increased 1 with intake amount increased 2g<br>>13g=6	<4g=0<br>4-5g=1<br>Score increased 1 with intake amount increased 2g<br>14g=6	<6g=0<br>6-7g=1<br>score increased 1 with intake amount increased 2g<br>>16g=6			
C7-Diet variety	(-12)-0	Diet variety	(-12)-0	≥12 kinds of food (soybean is 5g) =0; score decreased 1 with decreased 1 kinds of food						
C8-Drinking water	(-12)-0	Drinking water	(-12)-0	≥1200ml=0; score decreased 1 with intake amount decreased 100ml; <100ml=-12						

[1]This table has been reproduced from He, Y., et al., *Update of the Chinese diet balance index: DBI-16*. Acta Nutrimenta Sinica, 2018. 40(06): p. 526-530.

Supplementary Table S2. CHEI components and standard for scoring[1].

Component	Score		
	0	5	10
Adequacy			
Total Grains	0	≥2.5 SP/1000 kcal[2]	
Whole Grains and Mixed Beans	0	≥0.6 SP/1000 kcal	
Tubers	0	≥0.3 SP/1000 kcal	
Total Vegetables	0	≥1.9 SP/1000 kcal	
Dark Vegetables	0	≥0.9 SP/1000 kcal	
Fruits	0		≥1.1 SP/1000 kcal
Dairy	0	≥0.5 SP/1000 kcal	
Soybeans	0	≥0.4 SP/1000 kcal	
Fish and Seafood	0	≥0.6 SP/1000 kcal	
Poultry	0	≥0.3 SP/1000 kcal	
Eggs	0	≥0.5 SP/1000 kcal	
Seeds and Nuts	0	≥0.4 SP/1000 kcal	
Limitation			
Red Meat	≥3.5	≤0.4 SP/1000 kcal	
Cooking Oils	≥32.6		≤15.6 g/1000 kcal
Sodium	≥3608		≤1000 mg/1000kcal
Added Sugars	≥20%	≤10% of energy[3]	
Alcohol	≤60g (men)/40g (women)	≤60g (men)/40g (women)	

[1]This table has been reproduced from Yuan, Y.Q., et al., *The Development of a Chinese Healthy Eating Index and Its Application in the General Population*. Nutrients, 2017. 9(9).

[2]SP/1000 kcal: Standard Portion per 1000 kcal

[3]% of energy: percentage of energy

Supplementary Table S3. MDS components and standard for scoring[1].

Food group	Criteria for 1 point
Vegetables	Greater than median intake (servings/d)
Legumes	Greater than median intake (servings/d)
Fruit	Greater than median intake (servings/d)
Nuts	Greater than median intake (servings/d)
Whole grains	Greater than median intake (servings/d)
Red and processed meats	less than median intake (servings/d)
Fish	Greater than median intake (servings/d)
Ratio of monounsaturated to saturated fat	Greater than median intake (servings/d)
Ethanol	5-25 g/d

[1]This table has been reproduced from Fung, T.T., et al., *Diet-quality scores and plasma concentrations of markers of inflammation and endothelial dysfunction*. Am J Clin Nutr, 2005. 82(1): p. 163-73.

Supplementary Table S4. DASH score components and standard for scoring[1].

Component	Maximum score	Standard for maximum score	Standard for minimum score
Grains			
Total	5	≥6 servings/d	0 servings/d
High fiber	5	≥50% of daily servings	0% of daily servings
Vegetables	10	≥4 servings/d	0 servings/d
Fruit	10	≥4 servings/d	0 servings/d
Dairy			
Total	5	≥2 servings/d	0 servings/d
Low fat	5	≥75% of daily servings	0% of daily servings
Meat, poultry, fish, eggs	10	≤2 servings/d	≥4 servings/d
Nuts, seeds, legumes	10	≥4 servings/week	0 servings/week
Fats, oils	10	≤3 servings/d	≥6 servings/d
Sweets	10	≤5 servings/week	≥10 servings/week

[1]This table has been reproduced from Danielewicz, A., et al., Association of the Dietary Approaches to Stop Hypertension, Physical Activity, and Their Combination with Semen Quality: A Cross-Sectional Study. Nutrients, 2019. 12(1).

Supplementary Table S5. Associations between DBI-16 scores and cardiometabolic risk factors among 202 female participants with hyperlipidemia [1,2].

Cardiometabolic risk factors	DBI-TS		DBI-LBS		DBI-HBS		DQD	
	β coefficient (95% CI)	R^2	β coefficient (95% CI)	R^2	β coefficient (95% CI)	R^2	β coefficient (95% CI)	R^2
Lipid and lipoprotein profiles								
Triglyceride								
Model 1	-0.010 (-0.030, 0.010)	4.6%	0.017 (-0.008, 0.043)	5.0%	0.001 (-0.035, 0.038)	4.1%	0.030 (-0.018, 0.078)	4.8%
Model 2	-0.014 (-0.033, 0.006)	6.5%	0.017 (-0.009, 0.043)	6.4%	-0.015 (-0.056, 0.026)	5.9%	-0.014 (-0.033, 0.005)	5.8%
TC								
Model 1	0.011 (-0.003, 0.025)	13.1%	-0.006 (-0.024, 0.013)	12.2%	0.025 (0, 0.051)	13.7%	0.038 (0.004, 0.072)*	14.1%
Model 2	0.012 (-0.003, 0.026)	15.4%	-0.009 (-0.027, 0.010)	14.7%	0.027 (-0.002, 0.056)	15.7%	0.043 (0.002, 0.083)*	16.1%
LDL-C								
Model 1	0.007 (-0.009, 0.022)	8.1%	-0.001 (-0.021, 0.019)	7.8%	0.020 (-0.008, 0.049)	8.7%	0.040 (0.003, 0.078)*	9.7%
Model 2	0.008 (-0.008, 0.024)	9.9%	-0.003 (-0.023, 0.018)	9.5%	0.027 (-0.005, 0.059)	10.7%	0.055 (0.011, 0.100)*	12.1%
HDL-C								
Model 1	0.008 (0.001, 0.014)*	16.8%	-0.011 (-0.019, -0.003)*	17.6%	0.003 (-0.009, 0.014)	14.5%	-0.018 (-0.033, -0.003)*	16.6%
Model 2	0.008 (0.002, 0.014)*	19.2%	-0.012 (-0.020, -0.004)*	20.0%	0.005 (-0.008, 0.017)	16.7%	-0.024 (-0.042, -0.006)*	19.3%
TC : HDL-C ratio								
Model 1	-0.015 (-0.033, 0.003)	7.6%	0.028 (0.005, 0.051)*	8.9%	0.006 (-0.027, 0.039)	6.4%	0.066 (0.023, 0.110)*	10.3%
Model 2	-0.017 (-0.035, 0.001)	8.4%	0.028 (0.004, 0.052)*	9.3%	-0.001 (-0.039, 0.036)	6.9%	0.080 (0.029, 0.132)*	10.9%
LDL-C : HDL-C ratio								
Model 1	-0.010 (-0.024, 0.003)	7.1%	0.021 (0.004, 0.038)*	8.7%	0.009 (-0.016, 0.033)	6.3%	0.058 (0.026, 0.090)*	11.6%
Model 2	-0.010 (-0.024, 0.004)	7.2%	0.021 (0.003, 0.039)*	8.8%	0.010 (-0.018, 0.039)	6.6%	0.080 (0.042, 0.118)*	13.6%
ApoA1								
Model 1	0.004 (0, 0.008)*	10.3%	-0.005 (-0.010, -0.001)*	10.4%	0.003 (-0.004, 0.010)	8.5%	-0.008 (-0.017, 0.001)	9.5%
Model 2	0.005 (0.001, 0.008)*	13.1%	-0.006 (-0.011, -0.001)*	13.5%	0.003 (-0.004, 0.011)	11.0%	-0.013 (-0.024, -0.002)*	13.2%

ApoB								
Model 1	0.001 (-0.003, 0.005)	7.9%	0.002 (-0.004, 0.007)	8.1%	0.006 (-0.002, 0.013)	9.0%	0.016 (0.007, 0.026)*	12.8%
Model 2	0.001 (-0.003, 0.005)	9.5%	0.001 (-0.004, 0.006)	9.5%	0.005 (-0.003, 0.014)	10.2%	0.019 (0.008, 0.030)*	14.2%
ApoA1 : ApoB ratio								
Model 1	0 (-0.005, 0.006)	4.8%	-0.003 (-0.010, 0.004)	5.1%	-0.005 (-0.016, 0.005)	5.3%	-0.024 (-0.037, -0.011)*	10.5%
Model 2	0.001 (-0.005, 0.007)	5.1%	-0.003 (-0.010, 0.004)	5.4%	-0.005 (-0.016, 0.007)	5.4%	-0.031 (-0.046, -0.015)*	11.5%
Glucose homeostasis biomarkers								
Glucose								
Model 1	-0.002 (-0.018, 0.015)	7.9%	0.012 (-0.009, 0.033)	8.4%	0.019 (-0.011, 0.049)	8.5%	0.044 (0.005, 0.084)*	10.0%
Model 2	-0.002 (-0.018, 0.015)	9.0%	0.008 (-0.013, 0.030)	9.3%	0.014 (-0.020, 0.048)	9.3%	0.043 (-0.004, 0.091)	10.4%
HbA1c								
Model 1	0.002 (-0.009, 0.013)	6.1%	0.004 (-0.010, 0.018)	6.2%	0.014 (-0.006, 0.034)	6.9%	0.031 (0.005, 0.058)*	8.4%
Model 2	0.002 (-0.009, 0.013)	8.8%	0 (-0.014, 0.014)	8.8%	0.007 (-0.016, 0.029)	8.9%	0.022 (-0.009, 0.054)	9.6%
Blood pressure								
Systolic blood pressure								
Model 1	-0.090 (-0.330, 0.149)	15.6%	0.064 (-0.243, 0.371)	15.5%	-0.171 (-0.607, 0.265)	15.6%	-0.330 (-0.911, 0.251)	15.9%
Model 2	-0.062 (-0.303, 0.179)	17.3%	0.098 (-0.215, 0.412)	17.3%	-0.016 (-0.508, 0.476)	17.2%	-0.152 (-0.842, 0.538)	17.3%
Diastolic blood pressure								
Model 1	0.008 (-0.136, 0.151)	12.0%	-0.093 (-0.277, 0.091)	12.4%	-0.163 (-0.425, 0.098)	12.6%	-0.226 (-0.574, 0.123)	12.7%
Model 2	0.020 (-0.124, 0.164)	14.7%	-0.061 (-0.249, 0.126)	14.8%	-0.066 (-0.361, 0.228)	14.7%	-0.074 (-0.487, 0.339)	14.7%

[1] Data are presented as β coefficients (95% CI) per 1 SD of the DBI-16 score. TC, total cholesterol; LDL-C, low density lipoprotein cholesterol; HDL-C, high density lipoprotein; HbA1c, glycated hemoglobin A1c; Apo, apolipoprotein.

[2] Associations between the DBI-16 scores and cardiometabolic risk factors were analyzed using multivariable linear regression models. In model 1, the data were adjusted for potential confounders, including age, sex, BMI. Model 2 included model 1 plus additional sociodemographic and lifestyle confounders, including cigarette smoking, education status, physical activity and total energy intake. *$P < 0.05$.

Supplementary Table S6. Associations between DBI-16 scores and cardiometabolic risk factors among 67 male participants with hyperlipidemia [1,2].

Cardiometabolic	DBI-TS		DBI-LBS		DBI-HBS		DQD	
risk factors	β coefficient (95% CI)	R^2	β coefficient (95% CI)	R^2	β coefficient (95% CI)	R^2	β coefficient (95% CI)	R^2
Lipid and lipoprotein profiles								
Triglyceride								
Model 1	-0.052 (-0.098, -0.007)*	26.0%	0.051 (-0.002, 0.105)	24.4%	-0.041 (-0.121, 0.040)	21.5%	-0.072 (-0.186, 0.042)	22.2%
Model 2	-0.056 (-0.101, -0.011)*	34.0%	0.062 (0.011, 0.114)*	33.6%	-0.029 (-0.114, 0.055)	28.5%	-0.039 (-0.168, 0.089)	28.4%
TC								
Model 1	0.019 (-0.011, 0.049)	2.7%	-0.019 (-0.054, 0.015)	2.1%	0.014 (-0.038, 0.065)	0.8%	0.018 (-0.055, 0.092)	0.8%
Model 2	0.013 (-0.016, 0.042)	14.0%	-0.009 (-0.043, 0.024)	13.4%	0.019 (-0.034, 0.072)	13.7%	0.040 (-0.040, 0.120)	14.3%
LDL-C								
Model 1	0.027 (-0.004, 0.057)	8.1%	-0.028 (-0.064, 0.008)	7.3%	0.018 (-0.036, 0.072)	4.7%	0.040 (-0.036, 0.117)	5.6%
Model 2	0.022 (-0.009, 0.053)	14.9%	-0.021 (-0.056, 0.015)	14.0%	0.022 (-0.035, 0.078)	13.1%	0.055 (-0.030, 0.141)	14.4%
HDL-C								
Model 1	0.016 (0.005, 0.026)*	27.7%	-0.014 (-0.026, -0.001)*	23.5%	0.017 (-0.002, 0.036)	22.1%	0.005 (-0.023, 0.032)	18.7%
Model 2	0.016 (0.006, 0.027)*	29.7%	-0.015 (-0.028, -0.002)*	26.1%	0.017 (-0.003, 0.038)	23.7%	0 (-0.032, 0.032)	20.5%
TC : HDL-C ratio								
Model 1	-0.033 (-0.065, -0.002)*	28.1%	0.032 (-0.004, 0.068)	26.5%	-0.029 (-0.083, 0.026)	24.5%	-0.001 (-0.080, 0.077)	23.3%
Model 2	-0.044 (-0.072, -0.015)*	42.8%	0.045 (0.012, 0.079)*	41.4%	-0.031 (-0.086, 0.024)	36.5%	0.022 (-0.062, 0.106)	35.6%
LDL-C : HDL-C ratio								
Model 1	-0.007 (-0.032, 0.018)	9.2%	0.009 (-0.020, 0.038)	9.2%	-0.003 (-0.045, 0.040)	8.8%	0.033 (-0.028, 0.093)	10.3%
Model 2	-0.014 (-0.038, 0.010)	20.2%	0.017 (-0.011, 0.044)	20.3%	-0.004 (-0.049, 0.040)	18.8%	0.045 (-0.022, 0.112)	20.8%
ApoA1								
Model 1	0.007 (0.001, 0.014)*	21.7%	-0.006 (-0.014, 0.002)	18.5%	0.009 (-0.002, 0.020)	18.7%	0 (-0.017, 0.016)	15.7%
Model 2	0.008 (0.001, 0.015)*	23.6%	-0.007 (-0.015, 0.001)	20.6%	0.010 (-0.003, 0.022)	20.1%	-0.003 (-0.022, 0.017)	17.3%

ApoB								
Model 1	0.005 (-0.002, 0.013)	5.9%	-0.005 (-0.013, 0.003)	4.9%	0.005 (-0.008, 0.017)	3.6%	0.008 (-0.010, 0.026)	4.0%
Model 2	0.004 (-0.003, 0.011)	18.3%	-0.003 (-0.011, 0.005)	17.3%	0.006 (-0.006, 0.019)	17.9%	0.013 (-0.006, 0.032)	19.0%
ApoA1 : ApoB ratio								
Model 1	0.001 (-0.007, 0.010)	15.7%	-0.001 (-0.011, 0.09)	15.6%	0.003 (-0.012, 0.018)	15.7%	-0.009 (-0.029, 0.012)	16.4%
Model 2	0.003 (-0.005, 0.011)	25.9%	-0.003 (-0.013, 0.006)	25.8%	0.002 (-0.013, 0.017)	25.5%	-0.015 (-0.038, 0.008)	27.3%
Glucose homeostasis biomarkers								
Glucose								
Model 1	0.045 (0.014, 0.076)*	15.2%	-0.029 (-0.067, 0.008)	8.0%	0.069 (0.016, 0.123)*	13.1%	-0.033 (-0.113, 0.047)	5.7%
Model 2	0.045 (0.014, 0.077)*	19.0%	-0.028 (-0.065, 0.010)	12.2%	0.080 (0.022, 0.137)*	18.5%	-0.031 (-0.123, 0.060)	10.0%
HbA1c								
Model 1	0.014 (-0.006, 0.034)	12.7%	-0.006 (-0.029, 0.018)	10.6%	0.028 (-0.005, 0.062)	13.8%	-0.010 (-0.059, 0.039)	10.5%
Model 2	0.013 (-0.007, 0.033)	20.0%	-0.002 (-0.025, 0.021)	18.0%	0.038 (0.003, 0.073)*	23.2%	0.011 (-0.044, 0.066)	18.2%
Blood pressure								
Systolic blood pressure								
Model 1	0.125 (-0.346, 0.595)	13.5%	-0.128 (-0.673, 0.418)	13.5%	0.087 (-0.722, 0.895)	13.3%	-0.607 (-1.750, 0.535)	14.6%
Model 2	0.109 (-0.364, 0.582)	18.8%	-0.059 (-0.604, 0.486)	18.6%	0.213 (-0.648, 1.074)	18.8%	-0.411 (-1.716, 0.894)	19.0%
Diastolic blood pressure								
Model 1	-0.012 (-0.299, 0.275)	15.1%	-0.054 (-0.386, 0.279)	15.2%	-0.153 (-0.644, 0.337)	15.6%	-0.263 (-0.961, 0.435)	15.8%
Model 2	0.021 (-0.270, 0.311)	19.3%	-0.046 (-0.380, 0.288)	19.4%	0.046 (-0.574, 0.482)	19.3%	-0.025 (-0.827, 0.777)	19.3%

[1] Data are presented as β coefficients (95% CI) per 1 SD of the DBI-16 score. TC, total cholesterol; LDL-c, low density lipoprotein cholesterol; HDL-c, high density lipoprotein; HbA1c, glycated hemoglobin A1c; Apo, apolipoprotein.

[2] Associations between the DBI-16 scores and cardiometabolic risk factors were analyzed using multivariable linear regression models. In model 1, the data were adjusted for potential confounders, including age, sex, BMI. Model 2 included model 1 plus additional sociodemographic and lifestyle confounders, including cigarette smoking, education status, physical activity and total energy intake. *$P < 0.05$.

Supplementary Table S7. Associations between CHEI, MDS and DASH scores and cardiometabolic risk factors among 202 female participants with hyperlipidemia [1,2].

Cardiometabolic risk factors	CHEI		MDS		DASH scores	
	β coefficient (95% CI)	R^2	β coefficient (95% CI)	R^2	β coefficient (95% CI)	R^2
Lipid and lipoprotein profiles						
Triglyceride						
Model 1	-0.012 (-0.030, 0.007)	4.8%	-0.034 (-0.191, 0.123)	4.2%	-0.012 (-0.034, 0.009)	4.7%
Model 2	-0.014 (-0.033, 0.005)	6.7%	0.007 (-0.160, 0.174)	5.7%	-0.013 (-0.034, 0.009)	6.3%
TC						
Model 1	-0.004 (-0.018, 0.010)	12.2%	0.099 (-0.013, 0.211)	13.4%	0.005 (-0.011, 0.020)	12.2%
Model 2	-0.003 (-0.016, 0.011)	14.4%	0.134 (0.016, 0.252)	16.4%	0.007 (-0.009, 0.022)	14.7%
LDL-C						
Model 1	-0.004 (-0.018, 0.011)	7.9%	0.084 (-0.038, 0.207)	8.6%	0.004 (-0.013, 0.021)	7.8%
Model 2	-0.002 (-0.017, 0.013)	9.5%	0.099 (-0.031, 0.229)	10.5%	0.005 (-0.012, 0.022)	9.7%
HDL-C						
Model 1	0.003 (-0.003, 0.009)	14.8%	0.027 (-0.022, 0.076)	14.9%	0.005 (-0.002, 0.011)	15.1%
Model 2	0.004 (-0.002, 0.010)	17.1%	0.029 (-0.024, 0.081)	17.0%	0.005 (-0.002, 0.012)	17.4%
TC : HDL-C ratio						
Model 1	-0.012 (-0.029, 0.006)	7.2%	0.011 (-0.134, 0.155)	6.4%	-0.016 (-0.036, 0.004)	7.5%
Model 2	-0.013 (-0.031, 0.004)	7.8%	0.038 (-0.117, 0.193)	7.0%	-0.016 (-0.036, 0.004)	8.0%
LDL-C : HDL-C ratio						
Model 1	-0.007 (-0.020, 0.006)	6.7%	0.018 (-0.089, 0.126)	6.1%	-0.008 (-0.023, 0.007)	6.6%
Model 2	-0.007 (-0.020, 0.006)	6.8%	0.025 (-0.090, 0.140)	6.4%	-0.008 (-0.023, 0.007)	6.8%
ApoA1						
Model 1	0 (-0.003, 0.004)	8.2%	0.007 (-0.023, 0.037)	8.3%	0.002 (-0.002, 0.006)	8.6%
Model 2	0.001 (-0.003, 0.004)	10.8%	0.010 (-0.022, 0.042)	10.9%	0.003 (-0.001, 0.007)	11.4%

ApoB						
Model 1	-0.001 (-0.006, 0.003)	8.1%	0.016 (-0.017, 0.049)	8.2%	-0.001 (-0.005, 0.004)	8.0%
Model 2	-0.001 (-0.005, 0.003)	9.6%	0.022 (-0.012, 0.055)	10.2%	0 (-0.005, 0.004)	9.5%
ApoA1 : ApoB ratio						
Model 1	-0.001 (-0.006, 0.005)	4.8%	-0.006 (-0.051, 0.038)	4.8%	0.001 (-0.005, 0.007)	4.8%
Model 2	-0.001 (-0.006, 0.005)	5.1%	-0.014 (-0.062, 0.033)	5.3%	0.001 (-0.005, 0.007)	5.1%
Glucose homeostasis biomarkers						
Glucose						
Model 1	-0.007 (-0.022, 0.009)	8.1%	-0.132 (-0.261, -0.002)	9.6%	-0.015 (-0.033, 0.003)	9.1%
Model 2	-0.006 (-0.022, 0.010)	9.2%	-0.118 (-0.257, 0.020)	10.3%	-0.014 (-0.032, 0.004)	10.0%
HbA1c						
Model 1	-0.002 (-0.013, 0.008)	6.1%	-0.102 (-0.188, 0.016)	8.5%	-0.008 (-0.020, 0.004)	6.9%
Model 2	-0.001 (-0.012, 0.009)	8.8%	-0.081 (-0.173, 0.011)	10.1%	-0.007 (-0.019, 0.005)	9.3%
Blood pressure						
Systolic blood pressure						
Model 1	-0.056 (-0.285, 0.172)	15.5%	0.707 (-1.188, 2.602)	15.6%	-0.147 (-0.409, 0.115)	15.9%
Model 2	-0.034 (-0.264, 0.197)	17.2%	0.247 (-1.765, 2.260)	17.2%	-0.143 (-0.404, 0.118)	17.7%
Diastolic blood pressure						
Model 1	0.040 (-0.097, 0.178)	12.1%	0.556 (-0.581, 1.694)	12.4%	0.021 (-0.137, 0.179)	12.0%
Model 2	0.047 (-0.091, 0.184)	14.8%	0.218 (-0.985, 1.422)	14.7%	0.021 (-0.136, 0.178)	14.7%

[1] Data are presented as β coefficients (95% CI) per 1 SD of the CHEI, MDS, DASH score. TC, total cholesterol; LDL-c, low density lipoprotein cholesterol; HDL-c, high density lipoprotein; HbA1c, glycated hemoglobin A1c; Apo, apolipoprotein.

[2] Associations between the CHEI, MDS, DASH scores and cardiometabolic risk factors were analyzed using multivariable linear regression models. In model 1, the data were adjusted for potential confounders, including age, sex, BMI. Model 2 included model 1 plus additional sociodemographic and lifestyle confounders, including cigarette smoking, education status, physical activity and total energy intake. *$P < 0.05$.

Supplementary Table S8. Associations between CHEI, MDS and DASH scores and cardiometabolic risk factors among 67 male participants with hyperlipidemia [1,2].

Cardiometabolic risk factors	CHEI		MDS		DASH scores	
	β coefficient (95% CI)	R^2	β coefficient (95% CI)	R^2	β coefficient (95% CI)	R^2
Lipid and lipoprotein profiles						
Triglyceride						
Model 1	-0.021 (-0.056, 0.014)	22.0%	0.054 (-0.254, 0.362)	20.5%	-0.014 (-0.067, 0.039)	20.7%
Model 2	-0.026 (-0.062, 0.011)	30.0%	0.101 (-0.206, 0.408)	28.5%	-0.002 (-0.057, 0.053)	28.0%
TC						
Model 1	0.020 (-0.002, 0.043)	5.0%	-0.156 (-0.349, 0.036)	4.0%	-0.001 (-0.035, 0.033)	0.4%
Model 2	0.011 (-0.012, 0.034)	14.2%	-0.134 (-0.325, 0.056)	15.5%	-0.006 (-0.040, 0.029)	13.2%
LDL-C						
Model 1	0.025 (0.002, 0.048)*	10.0%	-0.168 (-0.369, 0.033)	7.8%	0.001 (-0.035, 0.036)	4.1%
Model 2	0.016 (-0.008, 0.041)	14.5%	-0.170 (-0.371, 0.032)	15.7%	-0.009 (-0.045, 0.028)	12.6%
HDL-C						
Model 1	0.004 (-0.005, 0.012)	19.3%	-0.015 (-0.088, 0.059)	18.7%	-0.001 (-0.013, 0.012)	18.6%
Model 2	0.004 (-0.005, 0.013)	21.4%	-0.016 (-0.093, 0.060)	20.7%	-0.002 (-0.016, 0.011)	20.6%
TC : HDL-C ratio						
Model 1	-0.001 (-0.025, 0.023)	23.3%	-0.126 (-0.333, 0.081)	24.9%	-0.009 (-0.045, 0.027)	23.5%
Model 2	-0.012 (-0.037, 0.012)	36.3%	-0.083 (-0.283, 0.118)	35.9%	-0.006 (-0.042, 0.030)	35.4%
LDL-C : HDL-C ratio						
Model 1	0.008 (-0.011, 0.027)	9.8%	-0.159 (-0.318, 0)*	13.7%	-0.006 (-0.034, 0.023)	9.0%
Model 2	-0.001 (-0.021, 0.018)	18.7%	-0.153 (-0.310, 0.005)	22.9%	-0.011 (-0.039, 0.018)	19.4%
ApoA1						
Model 1	0.001 (-0.004, 0.006)	15.9%	0.015 (-0.029, 0.059)	16.3%	0.002 (-0.006, 0.009)	16.0%
Model 2	0.002 (-0.004, 0.007)	17.8%	0.017 (-0.028, 0.063)	17.9%	0.002 (-0.006, 0.010)	17.5%

ApoB						
Model 1	0.006 (0.001, 0.011)*	9.6%	-0.037 (-0.084, 0.010)	6.3%	0.001 (-0.007, 0.009)	3.0%
Model 2	0.004 (-0.002, 0.009)	18.8%	-0.034 (-0.080, 0.011)	19.3%	0 (-0.009, 0.008)	16.8%
ApoA1 : ApoB ratio						
Model 1	-0.004 (-0.011, 0.002)	17.7%	0.044 (-0.011, 0.099)	18.5%	0.001 (-0.009, 0.010)	15.6%
Model 2	-0.002 (-0.009, 0.005)	25.7%	0.042 (-0.013, 0.096)	27.8%	0.002 (-0.008, 0.011)	25.5%
Glucose homeostasis biomarkers						
Glucose						
Model 1	0.005 (-0.020, 0.030)	5.0%	0.012 (-0.202, 0.226)	4.8%	-0.026 (-0.062, 0.010)	7.5%
Model 2	0.008 (-0.019, 0.034)	9.9%	0.073 (-0.146, 0.292)	10.0%	-0.018 (-0.057, 0.021)	10.6%
HbA1c						
Model 1	-0.003 (-0.018, 0.012)	10.5%	-0.051 (-0.181, 0.080)	11.0%	-0.013 (-0.036, 0.009)	12.1%
Model 2	-0.002 (-0.018, 0.014)	18.1%	-0.016 (-0.148, 0.116)	18.1%	-0.009 (-0.032, 0.015)	18.7%
Blood pressure						
Systolic blood pressure						
Model 1	-0.121 (-0.475, 0.233)	13.8%	-1.246 (-4.306, 1.814)	14.0%	-0.124 (-0.652, 0.405)	13.5%
Model 2	-0.166 (-0.542, 0.209)	19.4%	-0.859 (-3.981, 2.263)	18.9%	-0.023 (-0.582, 0.535)	18.5%
Diastolic blood pressure						
Model 1	-0.016 (-0.233, 0.200)	15.1%	-0.111 (-1.982, 1.760)	15.1%	0.128 (-0.193, 0.449)	15.9%
Model 2	0.020 (-0.211, 0.251)	19.3%	-0.111 (-2.027, 1.806)	19.3%	0.216 (-0.122, 0.554)	21.1%

[1] Data are presented as β coefficients (95% CI) per 1 SD of the CHEI, MDS, DASH score. TC, total cholesterol; LDL-c, low density lipoprotein cholesterol; HDL-c, high density lipoprotein; HbA1c, glycated hemoglobin A1c; Apo, apolipoprotein.

[2] Associations between the CHEI, MDS, DASH scores and cardiometabolic risk factors were analyzed using multivariable linear regression models. In model 1, the data were adjusted for potential confounders, including age, sex, BMI. Model 2 included model 1 plus additional sociodemographic and lifestyle confounders, including cigarette smoking, education status, physical activity and total energy intake. *$P < 0.05$.

Supplementary Table S9. Associations between DBI-16 scores and cardiometabolic risk factors among 269 Chinese participants with hyperlipidemia [1,2].

Cardiometabolic risk factors	DBI-TS		DBI-LBS		DBI-HBS		DQD	
	adjusted OR (95% CI)	Pseudo R^2	adjusted OR (95% CI)	Pseudo R^2	adjusted OR (95% CI)	Pseudo R^2	adjusted OR (95% CI)	Pseudo R^2
Lipid and lipoprotein profiles								
Triglyceride								
Model 1	0.965 (0.936,0.995)*	18.6%	1.052 (1.013,1.094)*	19.2%	0.989 (0.937,1.043)	16.3%	1.063 (0.986,1.146)	17.4%
Model 2	0.965 (0.934,0.997)*	26.3%	1.053 (1.011,1.097)*	27.0%	0.99 (0.927,1.057)	24.5%	1.098 (0.996,1.21)	25.9%
TC								
Model 1	1.032 (1.001,1.063) *	12.5%	0.964 (0.929,1.000)	12.3%	1.025 (0.972,1.08)	11.0%	1.034 (0.962,1.11)	11.0%
Model 2	1.040 (1.008,1.073) *	17.5%	0.958 (0.921,0.996) *	16.8%	1.051 (0.988,1.119)	15.8%	1.067 (0.976,1.166)	15.6%
LDL-C								
Model 1	1.030 (1.000,1.061) *	9.2%	0.962 (0.927,0.998) *	9.3%	1.016 (0.965,1.071)	7.5%	1.037 (0.967,1.113)	7.8%
Model 2	1.035 (1.004,1.067) *	13.3%	0.961 (0.925,0.999) *	12.9%	1.041 (0.979,1.107)	11.7%	1.091 (0.998,1.191)	12.7%
HDL-C								
Model 1	1.044 (1.011,1.077)*	26.0%	0.958 (0.921,0.997) *	24.9%	1.052 (0.995,1.113)	24.3%	0.961 (0.892,1.037)	23.5%
Model 2	1.044 (1.010,1.079)*	28.3%	0.956 (0.918,0.996) *	27.5%	1.061 (0.993,1.133)	26.8%	0.929 (0.844,1.022)	26.5%
TC : HDL-C ratio								
Model 1	0.955 (0.926,0.985)*	16.4%	1.068 (1.027,1.110)*	17.5%	0.985 (0.934,1.038)	12.5%	1.153 (1.065,1.248) *	18.4%
Model 2	0.948 (0.918,0.979)*	24.1%	1.069 (1.026,1.113)*	24.0%	0.956 (0.897,1.018)	20.4%	1.192 (1.079,1.316) *	25.0%
LDL-C: HDL-C ratio								
Model 1	0.972 (0.944,1.001)	10.4%	1.040 (1.002,1.079) *	10.7%	0.987 (0.937,1.04)	8.8%	1.095 (1.017,1.178) *	11.5%
Model 2	0.971 (0.941,1.001)	13.7%	1.038 (0.999,1.079)	13.7%	0.975 (0.917,1.036)	12.3%	1.128 (1.029,1.236) *	15.1%
ApoA1								
Model 1	1.050 (1.017,1.084)*	28.1%	0.952 (0.915,0.991)*	26.8%	1.058 (1.000,1.119) *	26.0%	0.950 (0.880,1.025)	25.1%

Model 2	1.052 (1.018,1.088) *	29.5%	0.948 (0.909,0.988) *	28.4%	1.073 (1.004,1.146) *	27.5%	0.911 (0.827,1.004)	27.2%
ApoB								
Model 1	1.017 (0.988,1.047)	4.0%	0.988 (0.954,1.025)	3.5%	1.031 (0.979,1.087)	4.0%	1.070 (0.996,1.149)	5.0%
Model 2	1.020 (0.990,1.051)	7.5%	0.988 (0.951,1.025)	6.9%	1.051 (0.989,1.117)	7.9%	1.123 (1.028,1.228) *	9.9%
ApoA1 : ApoB ratio								
Model 1	1.034 (1.004,1.064) *	10.2%	0.954 (0.919,0.990) *	10.9%	1.01 (0.958,1.064)	8.0%	0.889 (0.824,0.959) *	12.4%
Model 2	1.037 (1.005,1.070) *	16.8%	0.956 (0.920,0.994) *	16.6%	1.034 (0.972,1.100)	14.9%	0.872 (0.793,0.958) *	18.1%
Glucose homeostasis biomarkers								
Glucose								
Model 1	1.028 (0.998,1.059)	10.8%	0.979 (0.943,1.015)	9.8%	1.046 (0.991,1.103)	10.4%	1.029 (0.958,1.105)	9.5%
Model 2	1.029 (0.998,1.061)	13.4%	0.974 (0.938,1.012)	12.7%	1.051 (0.987,1.118)	13.0%	1.024 (0.938,1.119)	12.0%
HbA1c								
Model 1	0.995 (0.966,1.024)	7.0%	1.021 (0.985,1.059)	7.6%	1.028 (0.976,1.084)	7.5%	1.092 (1.015,1.175) *	9.6%
Model 2	0.991 (0.962,1.022)	12.7%	1.015 (0.977,1.055)	12.9%	1.005 (0.945,1.068)	12.6%	1.081 (0.988,1.182)	13.9%
Blood pressure								
Systolic blood pressure								
Model 1	0.977 (0.948,1.007)	21.5%	1.014 (0.976,1.054)	20.8%	0.954 (0.903,1.008)	21.7%	0.915 (0.848,0.987)	22.8%
Model 2	0.975 (0.945,1.007)	23.6%	1.019 (0.979,1.060)	23.0%	0.946 (0.887,1.009)	23.8%	0.889 (0.810,0.976)	25.2%
Diastolic blood pressure								
Model 1	0.988 (0.958,1.018)	18.8%	1.008 (0.970,1.047)	18.6%	0.977 (0.925,1.031)	18.8%	0.968 (0.899,1.042)	18.8%
Model 2	0.987 (0.956,1.019)	25.0%	1.016 (0.976,1.058)	25.0%	0.988 (0.926,1.054)	24.8%	0.990 (0.903,1.085)	24.8%

[1] Data are presented as adjusted OR (95% CI) per 1 SD of the DBI-16 score. TC, total cholesterol; LDL-c, low density lipoprotein cholesterol; HDL-c, high density lipoprotein; HbA1c, glycated hemoglobin A1c; Apo, apolipoprotein.

[2] Associations between the DBI-16 scores and cardiometabolic risk factors were analyzed using logistic regression models. In model 1, the data were adjusted for potential confounders, including age, sex, BMI. Model 2 included model 1 plus additional sociodemographic and lifestyle confounders, including cigarette smoking, education status,

physical activity and total energy intake. *$P < 0.05$.

Supplementary Table S10. Associations between CHEI, MDS and DASH scores and cardiometabolic risk factors among 269 Chinese participants with hyperlipidemia [1,2].

Cardiometabolic risk factors	CHEI		MDS		DASH scores	
	adjusted OR (95% CI)	Pseudo R^2	adjusted OR (95% CI)	Pseudo R^2	adjusted OR (95% CI)	Pseudo R^2
Lipid and lipoprotein profiles						
Triglyceride						
Model 1	0.987 (0.96,1.014)	16.7%	1.023 (0.812,1.288)	16.3%	1.002 (0.971,1.036)	16.3%
Model 2	0.980 (0.952,1.009)	25.2%	0.973 (0.754,1.257)	24.4%	0.998 (0.965,1.033)	24.4%
TC						
Model 1	1.016 (0.989,1.044)	11.2%	1.292 (1.024,1.629) *	12.8%	1.028 (0.995,1.062)	11.9%
Model 2	1.021 (0.993,1.05)	15.7%	1.309 (1.017,1.684) *	16.7%	1.027 (0.993,1.062)	15.8%
LDL-C						
Model 1	1.023 (0.996,1.051)	8.7%	1.286 (1.023,1.615) *	9.6%	1.008 (0.976,1.040)	7.4%
Model 2	1.030 (1.002,1.059) *	13.1%	1.298 (1.018,1.656) *	13.0%	1.007 (0.975,1.040)	11.1%
HDL-C						
Model 1	1.004 (0.976,1.033)	23.1%	1.020 (0.802,1.296)	23.0%	0.987 (0.954,1.021)	5149.0%
Model 2	1.007 (0.978,1.037)	25.7%	1.060 (0.817,1.376)	25.7%	0.989 (0.956,1.024)	25.7%
TC: HDL-C ratio						
Model 1	0.990 (0.963,1.017)	12.7%	1.009 (0.804,1.265)	12.4%	1.007 (0.975,1.040)	12.5%
Model 2	0.987 (0.959,1.015)	20.0%	1.068 (0.832,1.371)	19.7%	1.005 (0.972,1.039)	19.6%
LDL-C: HDL-C ratio						
Model 1	0.996 (0.970,1.022)	8.8%	1.004 (0.803,1.254)	8.7%	1.009 (0.978,1.042)	8.9%
Model 2	0.997 (0.970,1.025)	12.0%	1.032 (0.811,1.314)	12.0%	1.008 (0.975,1.041)	12.1%

ApoA1						
Model 1	1.003 (0.975,1.032)	24.4%	1.026 (0.806,1.306)	24.4%	1.008 (0.974,1.042)	24.5%
Model 2	1.005 (0.976,1.034)	25.8%	1.045 (0.807,1.353)	25.8%	1.009 (0.975,1.044)	25.9%
ApoB						
Model 1	1.014 (0.988,1.04)	3.8%	1.061 (0.854,1.319)	3.5%	1.009 (0.978,1.041)	3.5%
Model 2	1.018 (0.991,1.046)	7.4%	1.074 (0.849,1.358)	6.8%	1.009 (0.977,1.042)	6.8%
ApoA1 : ApoB ratio						
Model 1	1.002 (0.976,1.029)	7.9%	1.002 (0.802,1.251)	7.9%	1.002 (0.971,1.034)	7.9%
Model 2	1.001 (0.974,1.029)	14.4%	0.959 (0.751,1.224)	14.4%	1.005 (0.973,1.039)	14.4%
Glucose homeostasis biomarkers						
Glucose						
Model 1	1.011 (0.984,1.038)	9.5%	0.855 (0.682,1.071)	10.1%	0.979 (0.947,1.011)	10.0%
Model 2	1.014 (0.987,1.042)	12.3%	0.864 (0.678,1.100)	12.5%	0.979 (0.948,1.012)	12.6%
HbA1c						
Model 1	1.002 (0.977,1.029)	7.0%	0.716 (0.569,0.902) *	10.9%	0.980 (0.949,1.012)	7.7%
Model 2	1.007 (0.980,1.035)	12.7%	0.746 (0.582,0.956) *	15.1%	0.982 (0.95,1.015)	13.1%
Blood pressure						
Systolic blood pressure						
Model 1	0.979 (0.951,1.007)	21.5%	1.059 (0.837,1.341)	20.6%	0.980 (0.947,1.013)	21.2%
Model 2	0.974 (0.946,1.004)	23.9%	1.057 (0.820,1.361)	22.7%	0.978 (0.945,1.013)	23.3%
Diastolic blood pressure						
Model 1	0.981 (0.955,1.009)	19.3%	0.968 (0.766,1.223)	18.5%	0.984 (0.952,1.017)	18.9%
Model 2	0.983 (0.955,1.012)	25.3%	0.916 (0.706,1.189)	24.9%	0.980 (0.946,1.016)	25.2%

[1] Data are presented as adjusted OR (95% CI) per 1 SD of the CHEI, MDS, DASH score. TC, total cholesterol; LDL-c, low density lipoprotein cholesterol; HDL-c, high density lipoprotein; HbA1c, glycated hemoglobin A1c; Apo, apolipoprotein.

[2] Associations between the CHEI, MDS, DASH scores and cardiometabolic risk factors were analyzed using logistic regression models. In model 1, the data were adjusted for potential confounders, including age, sex, BMI. Model 2 included model 1 plus additional sociodemographic and lifestyle confounders, including cigarette smoking, education status, physical activity and total energy intake. $^*P < 0.05$.
