# Supplementary material for: Associations between Adherence to Four A Priori Dietary Indexes and Cardiometabolic Risk Factors among Hyperlipidemic Patients
Source: Nutrients. 2021 Jun 24;13(7):2179. doi: 10.3390/nu13072179 (PMC8308401; doi:10.3390/nu13072179)

Figure S1-Online Supplementary Figure. Flow diagram of recruitment and screening for participants with hyperlipidemia.

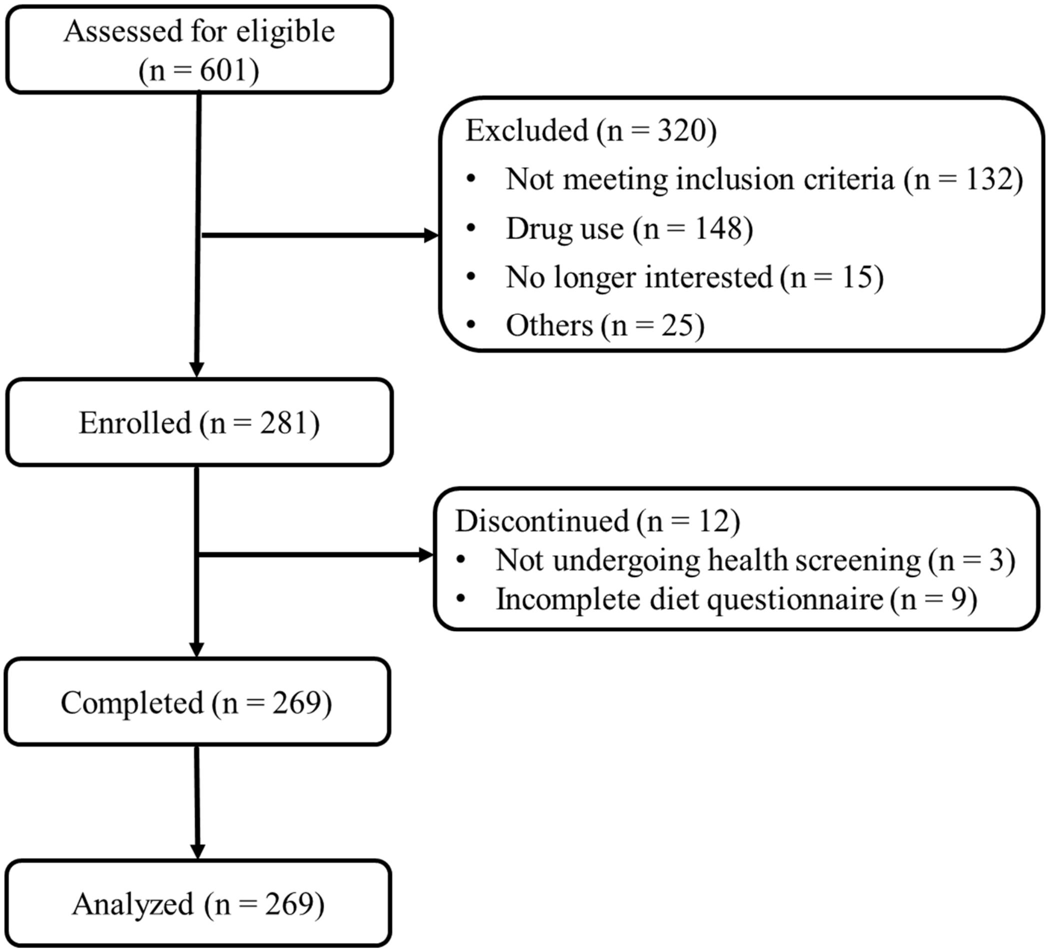

Supplement: Supplementary file 1 [file nutrients-13-02179-s001.zip › Supplementary Figure.pdf]
